# Unravelling the Surface Oxidation-Induced Evolution of the Electronic Structure of Gallium

**DOI:** 10.1021/acsami.3c09324

**Published:** 2023-09-29

**Authors:** Tzung-En Hsieh, Johannes Frisch, Regan G. Wilks, Marcus Bär

**Affiliations:** † Department Interface Design, Helmholtz-Zentrum Berlin für Materialien und Energie GmbH (HZB), 12489 Berlin, Germany; ‡ Department of Chemistry and Pharmacy, Friedrich-Alexander-Universität Erlangen-Nürnberg (FAU), 91058 Erlangen, Germany; § Energy Materials In situ Laboratory Berlin (EMIL), HZB, 12489 Berlin, Germany; ∥ Department X-ray Spectroscopy at Interfaces of Thin Films, Helmholtz Institute Erlangen-Nürnberg for Renewable Energy (HI ERN), 12489 Berlin, Germany

**Keywords:** gallium, gallium oxide, PES, IPES, valence band maximum, conduction band minimum

## Abstract

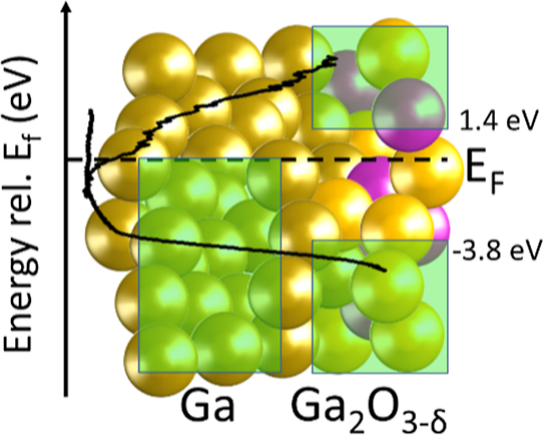

Gallium is widely used in liquid metal catalyst fabrication, and its oxidized species is a well-known dielectric material. In the past decades, these two species have been well studied separately. However, the surface oxide layer-induced impact on the chemical and electronic structure of (liquid) gallium is still mostly unclear because of the extreme fast formation of thermodynamically stable surface Ga_2_O_3_. In this study, we used a combination of direct and inverse photoemission complemented by scanning electron microscopy to examine the surface properties of Ga and Ga oxide (on a SiO_*x*_/Si support) and the evolution of the surface structure upon stepwise oxidation and subsequent reduction at an elevated temperature. We find oxidation time-dependent self-limited formation of a substoichiometric Ga_2_O_3−δ_ surface layer on the Ga nanoparticles. The valence band maximum (conduction band minimum) for this Ga_2_O_3−δ_ is located at −3.8 (±0.1) eV [1.4 (±0.2) eV] with respect to the Fermi level, resulting in an electronic surface band gap of 5.2 (±0.2) eV. Upon annealing in ultrahigh vacuum conditions, the Ga_2_O_3−δ_ surface layer can efficiently be removed when using temperatures of 600 °C and higher. This study reveals how the surface properties of Ga nanoparticles are influenced by stepwise oxidation–reduction, providing detailed insights that will benefit the optimization of this material class for different applications.

## Introduction

In recent decades, the exploitation and development of liquid metal materials have been an emerging research topic due to the wide range of applications. Liquid metals demonstrate advantages related to both characteristics—metallicity and liquidity—with high conductivity and a highly dynamic surface resulting in a large degree of flexibility.^1–5^ Bismuth (Bi), mercury (Hg), and gallium (Ga) are the liquid metal materials most often exploited as matrices for alloy material synthesis.^6,7^ For example, bimetallic Hg–Pd-based compounds are proven, efficient catalysts for phenol reduction, and Bi-based materials are utilized in thermal interface materials fabrication.^8,9^ However, the high toxicity of Hg and the high temperature required for Bi-alloy synthesis are drawbacks, particularly when considering large-scale industrial applications. Less toxic than Hg and with a much lower melting point than Bi (melting point of Ga = 30 °C ≪ melting point of Bi = 272 °C) Ga is a promising liquid metal candidate material. Ga-based liquid metals with a surface passivating gallium oxide shell are utilized in several applications, e.g., molecular electronics and thermoelectrics^10–13^ as well as thermal indicators for drug delivery.^14,15^ Recently, it has also been frequently employed as a matrix for liquid metal catalyst developments. The high dispersity of transition metals in Ga was observed for several Ga_*x*_M_*y*_ composites (M: Pt, Pb, Rh) which led to the development of supported catalytically active liquid metal solution (SCALMS).^3–5,16–18^ SCALMS is a new class of catalysts, which often comprises a liquid metal matrix (Ga) containing diluted metal active sites (M).

However, the high affinity for oxygen makes Ga easily turn into solid-state, high melting point gallium oxide, which will passivate (poison) the surface and fix the surface structure. Thus, Ga-based liquid metal alloys are often reported to have surfaces dominated by gallium oxide.^19,20^ However, a recent publication claims that peripheral Ga_2_O_3_ can also act as a matrix for catalytically active sites.^19^ Thus, studying the impact of oxide formation at the surface of liquid metal catalysts on their electronic structure is a crucial step for taking full advantage of this effect and using it for deliberate catalyst improvement.

In this study, we will use direct and inverse photoemission to shed light on the evolution of the chemical and electronic properties of Ga as a function of the degree of surface oxidation, providing new insights into the surface structure of SCALMS, unraveling the true state of its surface during catalytic reactions. In addition, the results may also be important for the development and application of Ga_2_O_3_ as viable high-*k* dielectric, transparent conductive oxide, and passivation layer candidate material.

## Experimental Section

### Materials and Sample Preparation

Gallium was purchased from Sigma-Aldrich (99.99999%). The oxygen for the surface oxidation experiments was purchased from Air Liquide Deutschland GmbH (99.998%). A polished p-type (Boron doped, Czochralsky tech. prepared, 2–4 Ω·cm resistance) silicon wafer having a natively formed silicon oxide on its surface is employed as substrate. All Ga samples were prepared via physical vapor deposition (PVD) using a SPECS EBE-4 e-beam evaporator under UHV condition (base pressure < 1 × 10^–8^ mbar). A deposition rate of 8 Å/min as established in previous experiments has been used for Ga deposition and is controlled via a quartz crystal microbalance before sample preparation. The target thickness of the deposited Ga films was 30 nm. The surface oxidation experiments were conducted in the same chamber as the sample preparation by means of stepwise exposing the Ga sample to 1 × 10^–6^ mbar of oxygen resulting in an accumulated oxidation time of 10, 30, 60, and 240 min while keeping the sample at room temperature. Note that the reproducibility of this sequential oxidation experiment was confirmed by repeating it three times, always resulting in similar findings. After surface oxidation at an oxygen partial pressure, one Ga sample was placed in ambient air for 1 month to form a thick oxide film. After oxidation in ambient air, the sample was transferred back into UHV to conduct photoemission measurements.

For the annealing experiment, we used one Ga/SiO_*x*_/Si sample that had been oxidized in 1 × 10^–6^ mbar of oxygen for 240 min and performed 30 min annealing steps at 3 × 10^–9^ mbar at temperatures of 400, 500, 600, 650, and 700 °C. After each step (i.e., deposition, oxidation, and annealing), the sample was transferred under UHV conditions to the surface analysis chamber (base pressure ≈ 2 × 10^–9^ mbar) for the room-temperature direct and inverse photoemission experiments, avoiding air exposure of the samples.

Fully exploiting the synthesis and characterization capacities of the interconnected system of different vacuum chambers in the Energy Materials In Situ Laboratory Berlin (EMIL),^21^ sample preparation as well as the oxidation/annealing treatments and spectroscopic measurements have been performed without exposing the sample to ambient conditions to avoid undesired surface oxidation and contamination of the PVD-deposited Ga samples by air and/or moisture.

### Direct Photoemission Spectroscopy

X-ray (XPS) and ultraviolet (UPS) photoelectron spectroscopy measurements were conducted using a nonmonochromatized Mg K_α_ (1253.56 eV) using a SPECS XR 50 X-ray source and the He II (40.8 eV) line using a Prevac UVS 40A2 gas discharge lamp, respectively. The photoelectrons were detected by a ScientaOmicron Argus CU electron analyzer. The pass energy for the core level detail spectra measurements was set to 20 eV, resulting in a total experimental energy resolution of approximately 1.2 eV for Mg K_α_-XPS. For the He II-UPS measurements, a pass energy of 5 eV was used, resulting in a total experimental resolution of 0.2 eV (see Supporting Information, for more details). The binding energy (BE) of the XPS and UPS measurements was calibrated by referencing the Fermi edge (*E*_F_) of the metallic Ga in the samples to a binding energy (BE) of 0 eV.

### Inverse Photoemission Spectroscopy

Inverse photoelectron spectroscopy (IPES) measurements were performed using a Kimball Physics Inc. EGPS-1022E electron gun with a BaO-coated filament, emitting electrons with a kinetic energy in the range of 5–15 eV. The OmniVac IPES1000 channeltron detector was utilized to detect the emitted photons. All IPES spectra are calibrated by setting the energy position of *E*_F_ measured on a clean Au foil to a binding energy of 0 eV. The total experimental resolution of the IPES setup has been determined to be 1.3 eV (see Supporting Information for more details).

### Scanning Electron Microscopy

Selected samples were examined by scanning electron microscopy (SEM) measurements using a Hitachi S 4100. For this, the sample to be studied is transferred under ambient conditions from the sample preparation chamber to the SEM setup. The SEM images were processed via ImageJ for particle size distribution analysis. For the size distribution determination, up to 200 particles in one SEM image were selected and evaluated.

## Results and Discussion

### Evolution of the Chemical Surface Structure upon Oxidation

The stepwise oxidation of the in-system prepared Ga samples was monitored by XPS. XPS is a surface-sensitive technique with an information depth that is governed by the photoelectrons’ inelastic mean free paths (IMFP, varying between 0.5 and 3 nm in Ga and Ga_2_O_3_ when using Mg K_α_ excitation).^22–24^ Hence, XPS is well-suited to study surface effects like oxidation. The XPS survey spectra of the in-system prepared Ga/SiO_*x*_/Si sample before and after stepwise oxidation are depicted in Figure S1 of the Supporting Information. The survey spectra are dominated by Ga-related core level and Auger lines as expected. No C 1s signal (expected at around 285 eV BE—see also Figure S2) can be observed confirming the benefit of in-system preparation and characterization and the high quality (i.e., contamination free condition) of the samples. Note that the spectrum of the sample oxidized in ambient conditions does exhibit a significant C 1s peak (see Figure S2), indicating surface contamination during the exposure to ambient conditions.

**Figure 1:**
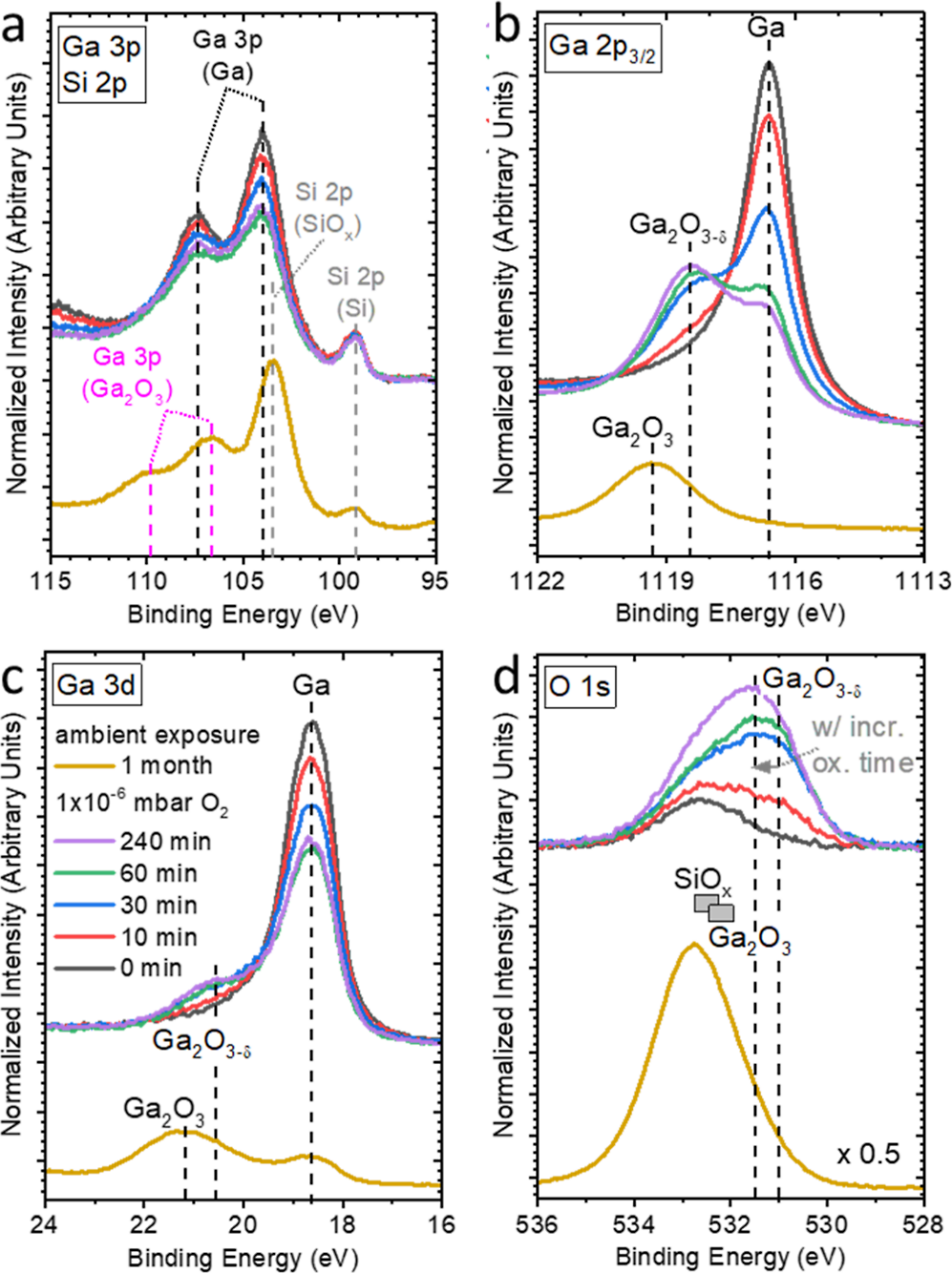
(a) Ga 3p/Si 2p, (b) Ga 2p_3/2_, (c) Ga 3d, and (d) O 1s Mg K_α_-XPS detail spectra of Ga oxidized at 1 × 10^–6^ mbar O_2_ for 0 min (black), 10 min (red), 30 min (blue), 60 min (green), 240 min (purple), and oxidized in ambient condition for 1 month (yellow). The boxes in panel (d) indicate the O 1s position for SiO_*x*_ and Ga_2_O_3_; for further peak identification, please see discussion in main text.

Close inspection of the detail spectra of the Ga 3p line (see Figure 1a—also depicting the Si 2p BE region), however, shows a Si 2p peak (of the SiO_*x*_/Si substrate) for all samples. Considering that the nominal thickness of the Ga layer by far exceeds the Si 2p photoelectrons’ IMFP (1.9 nm),^22–24^ this indicates an incomplete coverage of the SiO_*x*_/Si support by the deposited Ga. The degree of coverage is examined via quantitatively evaluating the Ga 3p and Si 2p core level peaks (see Figures S3 and S4), assuming that the Ga coverage of the SiO_*x*_/Si (where present) is thick enough to completely attenuate the Si 2p photoemission signal from the substrate, i.e., only the exposed (not covered) substrate regions are contributing to the Si 2p peak. The results indicate that the SiO_*x*_/Si support is covered to approximately 86% by the deposited Ga (see discussion in conjunction to Table S1 for more details) before oxidation. SEM measurements of an as-prepared Ga sample (see Figure S5 for an exemplary sample topography) indeed show the formation of spherical Ga nanoparticles (NPs) on the SiO_*x*_/Si support, corroborating the incomplete coverage; a wide, seemingly bimodal size distribution of Ga NPs in a range between 10 and 120 nm [resulting in an average NP size of (53 ± 30) nm] is observed (Figure S6). The broadening of the Ga 3p line observed with oxidation time in Figure 1a is attributed to an increasing contribution of oxidized Ga to the spectrum. For quantification of this chemical structure change, we use the narrower Ga 2p_3/2_ (at ≈1118 eV BE, Figure 1b) and Ga 3d (at ≈20 eV BE, Figure 1c) lines. Due to the very different BE (and, therefore, different IMFPs of the respective photoelectrons) of the considered Ga core levels [IMFP (Ga 2p_3/2_) = 0.6 nm ≪ IMFP (Ga 3d) = 2.7 nm], this also provides depth-dependent information on the chemical sample structure.^24,25^ The spectral shape of the Ga 2p_3/2_ and Ga 3d peaks for the in-system prepared Ga/SiO_*x*_/Si sample (black spectra in Figure 1b,c) as well as their BE positions (BE Ga 2p_3/2_ of 1116.7 eV and BE Ga 3d of 18.6 eV) are in line with the presence of one dominating chemical environment ascribed to metallic Ga;^26^ the detailed fit analysis of the Ga 2p_3/2_ and Ga 3d spectra is shown in Figures S7a and S8a, respectively. The attribution of the Ga 2p_3/2_ and Ga 3d lines to metallic Ga for the as-prepared sample is consistent with the observed dominance of the SiO_*x*_-related contribution (532.5 eV)^27^ to the O 1s spectrum for short oxidation times in Figures 1d and S9. With increasing exposure time to 1 × 10^–6^ mbar of O_2_, a spectral intensity increase at the high BE side of the Ga 2p_3/2_ and Ga 3d peaks can be observed, which we attribute to Ga–O bond formation. At the same time, the low BE O 1s peak contribution (at approximately 531 eV for short oxidation times) increases (see Figure 1d) together with the overall O 1s peak intensity (see XPS survey spectra in Figure S1). Detailed fit analysis (Figures S6 and S7) reveals the BE positions of these Ga oxide contributions to be at 1118.6 eV (Ga 2p_3/2_) and at 20.2 eV (Ga 3d) that is around 2 eV higher than the respective metallic Ga contributions, which is in agreement with previously reported data of thin Ga oxide formed on metallic Ga.^25^ The Ga/O stoichiometry of the formed gallium oxide is derived from quantitatively evaluating the Ga 3d and O 1s XPS data. For the Ga sample that had been oxidized in 1 × 10^–6^ mbar of O_2_ for 240 min (taking only the contributions of the O 1s and Ga 3d spectra assigned to Ga–O bonds into account), the Ga/O ratio is found to be 0.9 ± 0.1 (Table S2), which corresponds to a Ga_2_O_3−δ_ stoichiometry of Ga_2_O_2.3±0.2_. Note that even taking the total O 1s intensity into account, we derive an (upper bound) Ga/O ratio of 0.7 ± 0.1, corresponding to Ga_2_O_2.7±0.2_. In any case, the oxide layer that grows in 1 × 10^–6^ mbar of O_2_ on the metallic gallium particles exhibits a significant oxygen deficiency (compared to Ga_2_O_3_) suggesting the presence of oxygen vacancies. With increasing oxidation time in 1 × 10^–6^ mbar of O_2_, the intensity ratio between Ga 3p and Si 2p peaks varies, indicating the SiO_*x*_/Si coverage by Ga NPs decreases from 86 to 81% (Figure S3 and Table S1); attributed to an oxidation-induced dewetting of Ga layer on SiO_*x*_/Si substrate.

**Figure 2:**
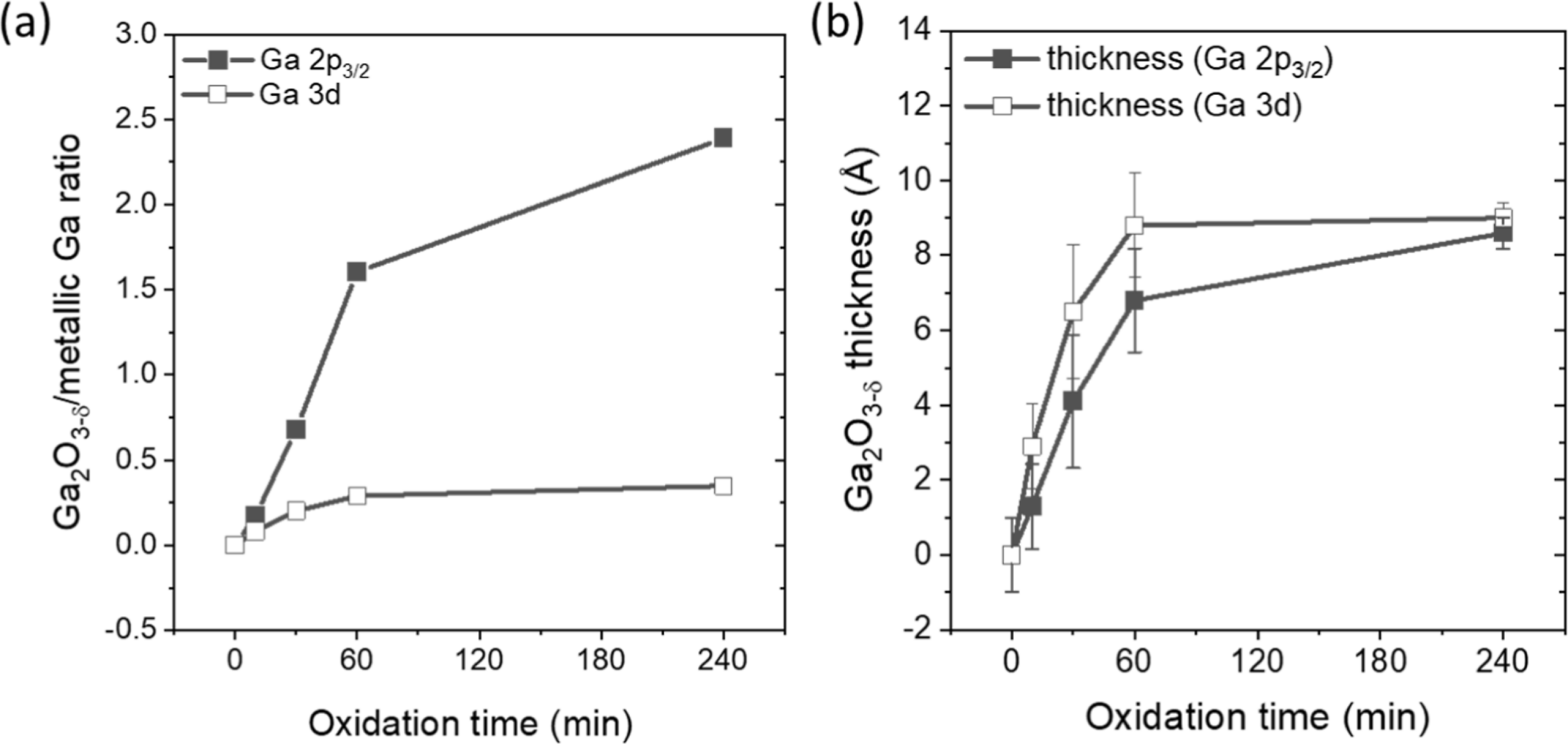
(a) Ga_2_O_3−δ_/metallic Ga ratio derived from the Ga 2p_3/2_ and Ga 3d XPS spectra measured for the Ga/SiO_*x*_/Si samples that have been oxidized in 1 × 10^–6^ mbar O_2_ for different times (0–240 min). (b) Corresponding Ga_2_O_3−δ_ thickness (details of film thickness determination is described in Supporting Information).

The spectral intensity attributed to Ga_2_O_3−δ_ increases faster with oxidation time for the more surface-sensitive Ga 2p_3/2_ line compared to the Ga 3d peak (see also discussion in conjunction with Figure 2a below), indicating that the oxidation predominantly takes place at the surface of the Ga particles. Note that the Ga 2p_3/2_ line (in contrast to the Ga 3d spectrum) of the sample oxidized in air for 1 month can basically be described by one species—again due to the higher surface sensitivity of the former. We find these Ga oxide contributions at higher BE (Ga 2p_3/2_: 1119.3 eV and Ga 3d: 21.2 eV, Figure 1b,c) compared to the Ga_2_O_3−δ_ contributions of the samples oxidized in 1 × 10^–6^ mbar of O_2_, and thus we attribute them to stoichiometric Ga_2_O_3_, in agreement with the literature (Ga 2p_3/2_ = 1119 ± 0.3 eV, Ga 3d = 20.8 ± 0.4 eV).^19,26,28^

The growth of Ga_2_O_3−δ_ is also observed in the O 1s XPS spectra (Figure 1d). We find the corresponding O 1s feature for the samples oxidized in 1 × 10^–6^ mbar of O_2_ at a lower binding energy (seemingly shifting from 531 to 531.5 eV with oxidation time) than that of the SiO_*x*_ contribution becoming more prominent with increasing oxygen exposure (as discussed above), eventually dominating the measured spectral region. The difference in O 1s peak position compared to typical Ga_2_O_3_ (532.2 eV)^29^ was also observed previously for thin Ga oxide layers formed on metallic Ga.^25^ For the sample oxidized in ambient conditions for 1 month, we find an O 1s line significantly increased in intensity (note the magnification factor in Figure 1d) and at a higher BE of 532.6 eV, i.e., a peak position that would be in agreement with the attribution to stoichiometric Ga_2_O_3_^29^ and/or SiO_*x*_ (532.5 ± 0.1 eV). The fit of the respective Ga 3p/Si 2p spectra shown in Figure 1a (see Figure S4) reveals a significant increase of the Si–O_*x*_ contribution to the Si 2p line together with more pronounced Ga oxide contribution to the Ga 3p line. While the latter is in agreement with the observation related to the corresponding Ga 2p_3/2_ and Ga 3d spectra in Figure 1b,c discussed above, we attribute the extraordinarily high Si–O_*x*_ contribution (especially when comparing to the ratio of the Si–O_*x*_/Si–Si contribution to the Si 2p line of the bare substrate—see Figure S3a) to a contamination of the sample surface while exposing the sample to ambient conditions presumably by a silicate. This is corroborated by the unreasonable low Ga/O ratio of 0.15 ± 0.1 (see Table S2) derived from the Ga–O contribution to the Ga 3d line and the O 1s peak intensity and the comparably low Ga line intensities in Figure 1a–c. As a result, we deliberately abstain from (quantitatively) interpreting the data of this sample.

How the surface oxidation evolves with time is represented in Figure 2a, showing the evolution of the Ga_2_O_3−δ_/metallic Ga ratio of the respective contributions to the Ga 2p_3/2_ and Ga 3d core levels. The Ga_2_O_3−δ_/metallic Ga ratio derived from the Ga 2p (Ga 3d) line increases rapidly from 0 to 1.6 (0 to 0.3) in the first hour of oxidation in 1 × 10^–6^ mbar of O_2_, while in additional 3 h, only an increase from 1.7 to 2.4 (0.3 to 0.35) can be observed. Note that the identification of a low BE feature in the O 1s spectrum (Figure S9) of the Ga/SiO_*x*_/Si sample before deliberate oxidation, presumably being ascribed to the initial presence of Ga–O bonds, is accounted for by adding a larger error bar to the “0 min” data point in Figure 2. The fact that Ga_2_O_3−δ_ contribution to the Ga 2p_3/2_ increases faster compared to the Ga_2_O_3−δ_ contribution of the Ga 3d line can be explained by the different BE, resulting in different IMFPs—making the Ga 2p_3/2_ data more surface sensitive then the Ga 3d data (see discussion above). Hence, the higher Ga_2_O_3−δ_ contribution in the Ga 2p_3/2_ spectra compared to that in the Ga 3d spectra for any given oxidation time is indicative for a Ga_2_O_3−δ_ formation that mainly takes place at the sample surface. The saturation of the oxidation rate then suggests that the rate-determining step of this Ga surface oxidation is the diffusion of oxygen into the increasingly buried Ga once the initial Ga_2_O_3−δ_ layer is formed at the surface with the growing Ga_2_O_3−δ_ increasingly suppressing any further O_2_ diffusion to the Ga.^20,30,31^ The thickness of the Ga_2_O_3−δ_ as derived based on the oxide/metal ratio derived from the fits of the corresponding core level contributions and assuming a simple overlayer model is shown in Figure 2b. More details on this analysis are described in Supporting Information. For the Ga/SiO_*x*_/Si that has been oxidized for 10 min, the oxide contribution to the Ga 2p_3/2_ (Ga 3d) line is in agreement with the formation of a 1.4 ± 1.1 Å (2.9 ± 1.1 Å) Ga_2_O_3−δ_ layer, which is less than 5.6 Å, i.e., the thickness of monolayer Ga_2_O_3_,^32^ indicating only a partially oxidized Ga surface. The formation of submonolayer gallium oxide layers under these conditions is consistent with previous studies.^11,12^ For the sample that has been oxidized for 60 min, the film thickness increased to 6.8 ± 1.4 Å (8.8 ± 1.4 Å), i.e., roughly equivalent or representing slightly more than a monolayer of Ga_2_O_3_.^18,32^ The final point, the sample after 240 min oxidation, shows a thickness of 8.6 ± 0.4 Å (9.1 ± 0.4 Å), indicating the oxygen-diffusion-limited formation of a second Ga oxide monolayer (Figure 2b, Tables S3 and S4). The oxide film thickness of the sample exposed to ambient oxidations for 1 month is estimated to be 35 ± 1 Å, indicating the formation of approximately 6 monolayers of Ga oxide at the sample surface (Table S5). Ga_2_O_3_ layers of similar thickness were reported to form in ambient conditions for Ga and GaRh samples with <1 at. % Rh.^5,19,33^ Note the observed difference of the film thickness values derived from the Ga 2p_3/2_ and Ga 3d core levels, respectively, can be attributed to the deviation of the assumed simple model (planar thin film overlayer) from the real sample (nanoparticulate core–shell structure). The details are discussed in the Supporting Information.

### Evolution of the Electronic Structure upon Oxidation

**Figure 3:**
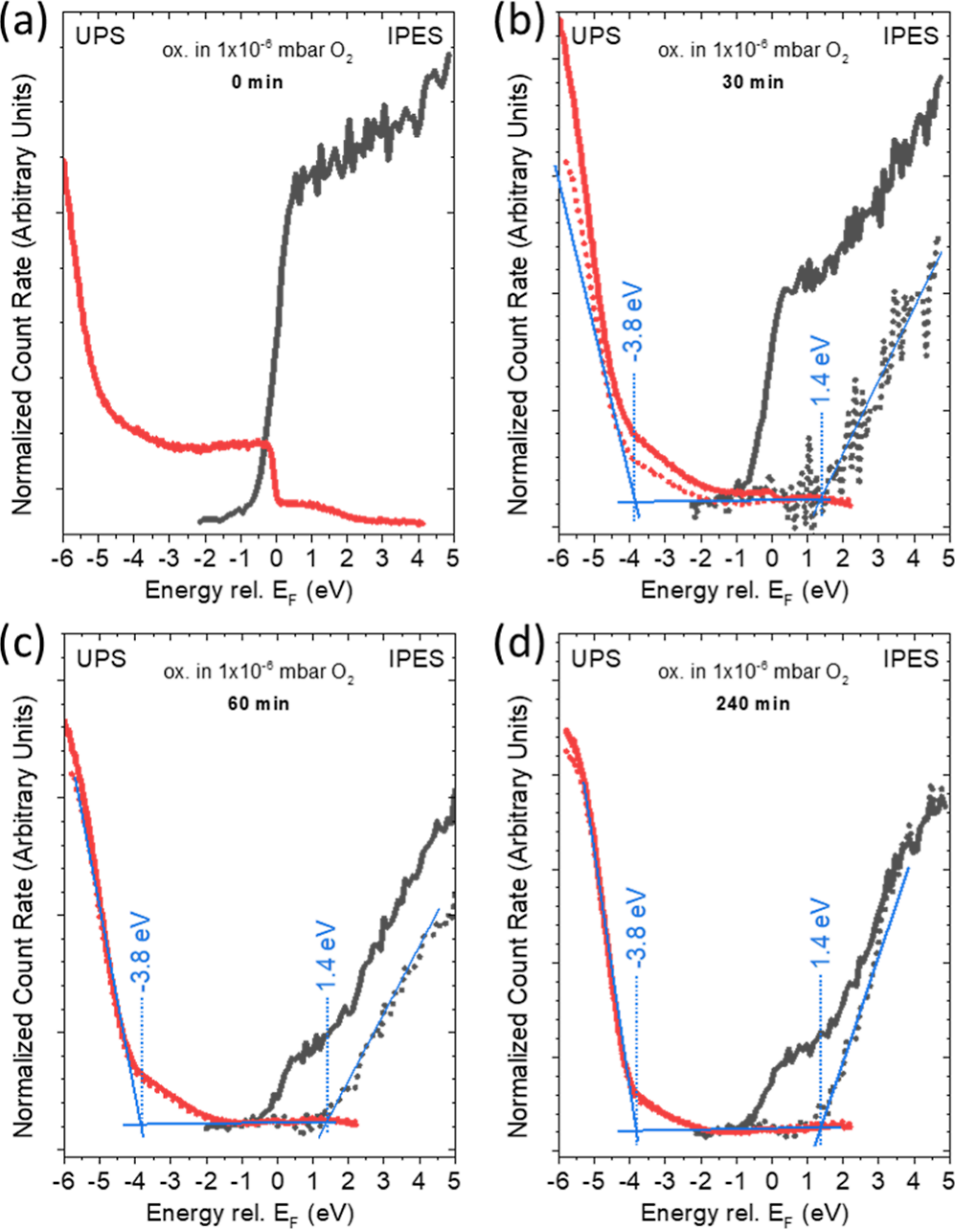
He II-UPS (red) and IPES (black) data (on a common energy scale with “0” indicating the position of the Fermi level) of a Ga/SiO_*x*_/Si sample before (a) and after 30 (b), 60 (c), and 240 min (d) exposure to 1 × 10^–6^ mbar O_2_. The linear extrapolation to derive VBM and CBM positions together with the derived values is also indicated. The VBM and CBM values were derived using the UPS and IPES spectra from which the metallic Ga contribution had been subtracted (dashed lines) and have an experimental uncertainty of ±0.1 and ±0.2 eV, respectively.

The evolution of the electronic structure of the occupied and unoccupied density of states upon surface oxidation is probed by UPS and IPES, respectively. Using the leading edges in the UPS and IPES spectra allows the determination of the valence band maximum (VBM) and conduction band minimum (CBM), respectively. In consequence, also the electronic surface band gap of the material can be derived: *E*_g_^Surf^ = CBM – VBM. Corresponding UPS and IPES data of the Ga/SiO_*x*_/Si samples before and after stepwise oxidation are shown in Figure 3, revealing insights on the electronic structure changes upon oxidation. For the as-prepared Ga/SiO_*x*_/Si sample, we find spectral intensity up to the Fermi level (*E*_F_) in both the UPS and IPES spectra, indicating a dominant metallic nature (*E*_g_^Surf^ = 0 eV) of the sample surface (Figure 3a), as expected. Upon oxidation and with increasing oxidation time, the spectral intensity in the vicinity of the Fermi level decreases, indicating an increasing impact of the formed Ga_2_O_3−δ_ at the sample surface on the electronic structure. This results in Ga_2_O_3−δ_-related VBM and CBM features significantly below or above *E*_F_, respectively, demonstrating the formation of a gap between the valence and conduction bands as expected for gallium oxide. The different surface sensitivities of UPS and IPES explain why the Fermi level-related spectral features disappear on a different (oxidation) time scale and, thus, oxide layer thickness (see Figure 2b). While the IMFP of *E*_F_-related photoelectrons in the UPS spectra (excited by He II, i.e., 40.8 eV) is around 4 Å,^34^ the electrons coupling into empty conduction band states as part of the IPES measurement process have a significantly lower (<10 eV) kinetic energy and, thus, according to the universal curve, are expected to have a larger IMFP.^24,35^ Thus, while *E*_F_-related spectral features can still be observed in the IPES spectra even for an oxidation time of 240 min (Figure 3d), these features have already almost completely disappeared in the UPS data of the sample that has only been oxidized for 60 min (Figure 3c). Hence (in this case), the more surface-sensitive UPS mainly probes the Ga_2_O_3−δ_ surface, while the more bulk-sensitive IPES is still able to detect a significant amount of metallic Ga buried under a closed film of Ga_2_O_3−δ_.

In order to separate the contributions of metallic Ga (resulting in spectral intensity around *E*_F_) and Ga_2_O_3−δ_ (resulting in VBM and CBM related spectral intensity below and above *E*_F_), suitably scaled UPS and IPES spectra of the as-prepared (not deliberately oxidized) Ga sample have been subtracted from the corresponding data of the oxidized samples (see dashed lines in Figure 3). Linear extrapolation of the leading edges of these UPS and IPES difference spectra has then been used to derive the VBM and CBM positions, respectively, with respect to *E*_F_. The thus derived VBM and CBM values of Ga_2_O_3−δ_ for oxidation times of 30 min and longer are −3.8 (±0.1) and 1.4 (±0.2) eV, respectively. Hence, the surface band gap of the Ga_2_O_3−δ_ surface layer is estimated to be around 5.2 (±0.2) eV. Note that there is a low/energy region (1.0–2.5 eV) in the IPES spectra which could, arguably, be used for the linear approximation of the leading edge. In that case, the obtained CBM would be closer to 1.1 eV, bringing the band gap closer to agreement with expected gap values reported for γ-Ga_2_O_3_.^36,37^ As the absolute position of the CBM does not significantly influence the analysis in our study, we will continue to use the more prominent region in Figure 3. However, within experimental uncertainty, we also find a fair agreement with the higher bound band gap values typically reported for β-Ga_2_O_3_^38^ (that approach, but usually are below 5 eV).

Note that despite this large band gap (which would be indicative for an electric insulator), oxygen-deficient gallium oxide with a significant concentration of oxygen vacancies (which are efficient n-type dopants in Ga_2_O_3_) can be highly conductive as reported in previous studies.^10–12,39^ As a matter of fact, the pronounced spectral intensity starting at around 2 eV above VBM in the UPS data for the stepwise oxidized Ga samples (see Figure S11 for the 240 min oxidized sample) is attributed to oxygen vacancy-related surface defect states often reported to be present in substoichiometric gallium oxide,^39,40^ in agreement with the derived oxygen deficiency observed for the formed Ga_2_O_3−δ_ layer in our study and justifying using the main edge in the UPS spectra for VBM determination. Note that the shape of this above-VBM intensity changes significantly for the sample that has been oxidized in ambient conditions (Figure S12) and we also observe the disappearance of the *E*_F_ feature in the corresponding IPES data. Whether this indicates the electronic structure of a thick stoichiometric Ga_2_O_3_ layer (with a significantly lower concentration of oxygen vacancy derived surface defect states) or is a result of sample contamination remains an open question. However, also for this sample, we find the same surface band gap as derived for the (contamination-free) samples oxidized in 1 × 10^–6^ mbar O_2_ (see Figure S13).

The interference of potentially present spectral features attributed to uncovered SiO_*x*_/Si support with those related to Ga_2_O_3−δ_ in the UPS and IPES spectra with respect to the determination of the VBM and CBM positions of Ga_2_O_3−δ_ can mainly be ruled out as any contribution can only be minor (coverage of the support is >80%) and would be expected further away from *E*_F_.^41^

### Surface Structure Evolution upon Annealing

**Figure 4:**
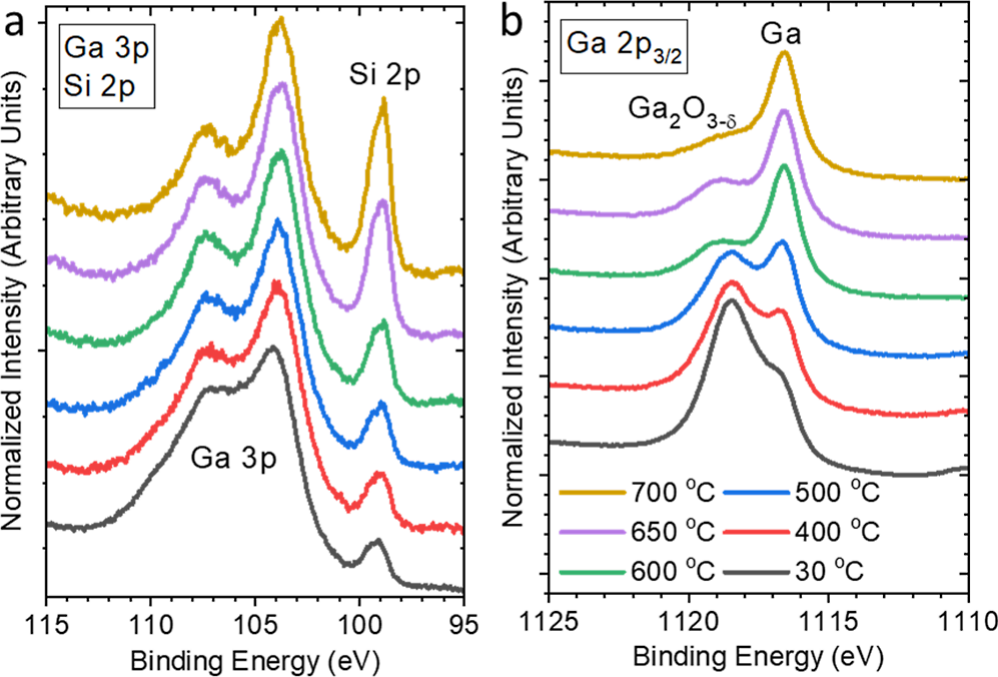
(a) Ga 3p/Si 2p and (b) Ga 2p_3/2_ Mg K_α_-XPS spectra of a Ga/SiO_*x*_/Si sample oxidized in 1 × 10^–6^ mbar of O_2_ for 240 min before and after annealing in UHV to different temperatures (400–700 °C). All measurements were taken at room temperature (i.e., after sample cool down).

**Figure 5:**
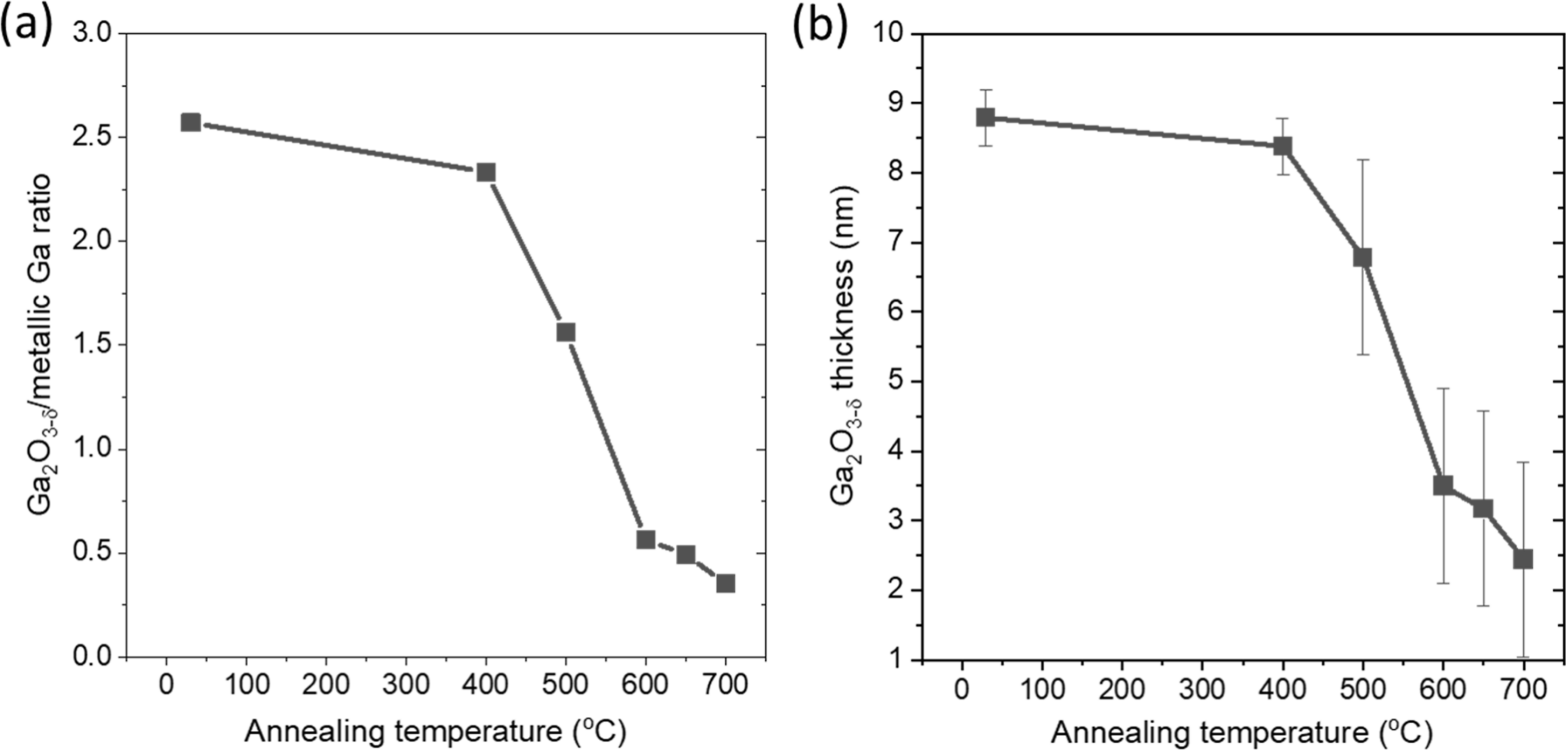
(a) Ga_2_O_3−δ_/metallic Ga ratio of the corresponding contributions to the Ga 2p_3/2_ XPS spectra measured for the Ga/SiO_*x*_/Si samples that have been oxidized in 1 × 10^–6^ mbar O_2_ for 240 min and annealed at temperatures between 400 and 700 °C. (b) Corresponding Ga_2_O_3−δ_ thicknesses (see Supporting Information for more details on the thickness determination).

Since gallium-based liquid metal alloys are utilized as catalysts at elevated reaction temperatures, we finally study the impact of different annealing treatments on the chemical and electronic structure of oxidized Ga/SiO_*x*_/Si samples. In the literature, it is reported that the gallium oxide surface layer is efficiently removed when the sample is heated to 600 °C, which can be attributed to formation of volatile Ga_2_O species desorbing from the Ga sample during annealing in UHV conditions.^42–44^ Such surface reduction was also observed in Ga-based metal alloys with extremely diluted transition metals (<1 at. %) in Ga.^5,19^ Thus, we conducted combined XPS and UPS measurements on one Ga/SiO_*x*_/Si sample that had been oxidized in 1 × 10^–6^ mbar of oxygen for 240 min and then successively annealed for 30 min to temperatures from 400 to 700 °C in UHV (3 × 10^–9^ mbar) to resolve the chemical and electronic structure variation caused by annealing at different temperatures (Figure 4). The Ga_2_O_3−δ_ feature in the Ga 3p and Ga 2p_3/2_ spectra is reduced after annealing (even at the lowest temperature of 400 °C), indicating a reduction of the previously oxidized Ga NPs. Quantitative analysis shows that the Ga_2_O_3−δ_/metallic Ga ratio has decreased from 2.6 to 2.3, and further decreases to 1.6 after 30 min annealing at 500 °C (Figures 5 and S14). The mainly unaffected intensity ratio of the Ga 3p and Si 2p core levels in this annealing temperature regime (see Figure 4a) indicates that the formed (and still present) Ga_2_O_3−δ_ surface layer prevents particle agglomeration-induced dewetting upon moderate annealing. When the sample is further annealed at 600 °C for 30 min, we observe a significant drop of the Ga_2_O_3−δ_ content to 0.6, indicating that the oxide layer is more efficiently removed. After additional subsequent annealing for 30 min to 700 °C the Ga_2_O_3−δ_ content drops further to 0.4 (Figures 5 and S14). Using the same analytical approach to derive the Ga_2_O_3−δ_ layer thickness as used above, we find that the thickness of Ga_2_O_3−δ_ decreases from (8.8 ± 0.4) Å to (2.4 ± 1.4) Å, i.e., nearly 73% of the surface Ga_2_O_3−δ_ has been removed at that point. Correspondingly, the Fermi-edge-related intensity in the UPS spectra is enhanced (see Figure S15), indicating that the metallic nature of Ga increasingly dominates the VB region again. However, residual Ga_2_O_3−δ_-related spectral features can still be observed in Ga 3p and (more prominently) in Ga 2p_3/2_ spectra (see Figure 4a,b), indicating that some Ga_2_O_3−δ_ remains. In this annealing temperature regime (i.e., ≥600 °C), the Si 2p peak intensity significantly increases (Figure 4a), which we attribute to substrate dewetting. Larger (agglomerated) particles can indeed be observed in corresponding SEM images (Figure S16). The size distribution analysis corroborates the particle enlargement resulting in an average NP size of (105 ± 61) nm (Figure S17), roughly doubling the NP size compared to pristine Ga particles. We speculate that the dewetting (particle agglomeration) is promoted by the existence of liquid Ga that is for annealing temperatures of ≥600 °C not surrounded by a solid Ga_2_O_3−δ_ shell.

## Conclusions

In this study, we unravel the surface-derived electronic and chemical properties of Ga nanoparticles on a SiO_*x*_/Si support and how they change upon stepwise oxidation and subsequent annealing induced reduction via XPS, UPS, and IPES. The surface oxidation-induced chemical and electronic structure variation is examined via oxidation time-dependent XPS measurements. UPS and IPES measurements are conducted to determine the impact of surface oxidation on the electronic structure, specifically the VBM and CBM positions and the electronic surface band gap. We find an oxidation time-dependent formation of a substoichiometric Ga_2_O_3−δ_ surface layer on the Ga nanoparticles when the sample is oxidized in 1 × 10^–6^ mbar O_2_, starting to level off at a thickness of around 9 Å when oxidized longer than 60 min, indicating a self-limiting (presumably diffusion controlled) oxidation mechanism. The VBM [CBM] for this Ga_2_O_3−δ_ is located at −3.8 (±0.1) eV [1.4 (±0.2) eV] with respect to the Fermi level, resulting in an electronic surface band gap of 5.2 (±0.2) eV. Upon annealing in 1 × 10^–9^ mbar UHV conditions, the Ga_2_O_3−δ_ starts to slowly being reduced at a temperature of 400 °C. At temperatures of 600 °C and higher, the Ga_2_O_3−δ_ contribution decreases significantly faster and a substrate dewetting can be observed. The annealing-induced removal of the Ga_2_O_3−δ_ shell from the Ga nanoparticles also manifests in the reappearance of the spectral photoemission feature related to the Fermi level, indicating that upon this reduction treatment, the metallic nature of Ga increasingly dominates the electronic structure again as well. This study provides detailed information on how the chemical and electronic structure of Ga nanoparticles is impacted and can deliberately be tuned by stepwise oxidation/reduction paving the way for an insight-driven optimization of related applications in the field of liquid metal catalysts and dielectric materials.

## Supporting Information Available

Description of procedure of XPS peak analysis, SEM characterizations, additional XPS/UPS spectra and fitting results including quantification (PDF)
