# Supplementary material for: Unravelling the Surface Oxidation-Induced Evolution of the Electronic Structure of Gallium
Source: ACS Appl Mater Interfaces. 2023 Sep 29;15(40):47725–32. doi: 10.1021/acsami.3c09324 (PMC10571040; doi:10.1021/acsami.3c09324)
Supplement: Supplementary file 1 — am3c09324_si_001.pdf [file am3c09324_si_001.pdf]

Supporting Information

Unravelling the Surface Oxidation Induced Evolution of the Electronic Structure of Gallium

Tzung-En Hsieh[1*], Johannes Frisch[1,3], Regan G. Wilks[1,3], Marcus Bär[1,2,3,4*]

[1]*Department Interface Design, Helmholtz-Zentrum Berlin für Materialien und Energie GmbH (HZB), 12489 Berlin, Germany*

[2]*Department of Chemistry and Pharmacy, Friedrich-Alexander-Universität Erlangen-Nürnberg (FAU), 91058 Erlangen, Germany*

[3]*Energy Materials In-situ Laboratory Berlin (EMIL), HZB, 12489 Berlin, Germany*

[4]*Department X-ray Spectroscopy at Interfaces of Thin Films, Helmholtz Institute Erlangen-Nürnberg for Renewable Energy (HI ERN), 12489 Berlin, Germany*

Corresponding Author

Tzung-En Hsieh: tzung-en.hsieh@helmholtz-berlin.de

Marcus Bär: marcus.baer@helmholtz-berlin.de

Table of Contents:

XPS analysis S3
Determination of $Ga_2O_{3-\delta}$ film thickness S5
Figure S1. Survey spectra of Ga particles after different oxidation time S7
Figure S2. C 1s spectra of Ga particles after different oxidation time S8
Figure S3. Ga 3p/Si 2p core level fitting results of SiO_x/Si support and Ga NPs on SiO_x/Si S9
Figure S4. Ga 3p/Si 2p core level fitting results of Ga NPs after 1-month oxidation at ambient S10
Figure S5. SEM image of as prepare Ga NPs on SiO_x/Si substrate S11
Figure S6. Size distribution of Ga NPs on SiO_x/Si substrate S12
Figure S7. Ga $2p_{3/2}$ core level fitting results of Ga after oxidation S13
Figure S8. Ga 3d core level fitting results of Ga after oxidation S14
Figure S9. O 1s core level fitting results of as prepared Ga NPs S15
Figure S10. O 1s core level fitting results of Ga NPs after oxidation at elevated conditions S16
Figure S11. Examination of surface defect states in valence band S17
Figure S12. UPS/IPES spectra of Ga NPs after surface oxidation at elevated conditions S18
Figure S13. He-I UPS of Ga NPs before and after surface oxidation at elevated conditions S19
Figure S14. Ga$2p_{3/2}$ core level fitting results of Ga after annealing under elevated temperature S20
Figure S15. He-II UPS spectra of oxidized Ga NPs annealed at elevated temperature S21
Figure S16. SEM images of as Ga on SiO_x substrate before and after 700 ^oC annealing S22
Figure S17. Size distribution of 700 ^oC annealed Ga particles on SiO_x/Si substrate S23
Table S1. Examination of surface coverage of Ga NPs on SiO_x/Si support S24
Table S2. Examination of Ga/O stoichiometry of oxidized Ga NPs S25
Table S3. Examination of $Ga_2O_{3-\delta}$ layer thickness on Ga 2p core level fitting S26
Table S4. Examination of $Ga_2O_{3-\delta}$ layer thickness by Ga 3d core level fitting S26
Table S5. Thickness of $Ga_2O_{3-\delta}$ layer on Ga particle after 1-month ambient oxidation S27
References S28

XPS analysis

Energy resolution

In all XPS measurements with Mg K_a source, the pass energy for the core level detail spectra measurements was set to 20 eV, resulting in a total energy resolution of approximately 1.2 eV. For the He II-UPS measurements, a pass energy of 5 eV was used, resulting in a total energy resolution of 0.2 eV. The total energy resolution of the IPES setup is determined as 1.3 eV via fitting of a measured Fermi-edge (E_F) of a clean gold film by the following fit function with correction of temperature (T = 300 K) employing the Boltzmann constant ($K_b \cdot T$ = 25 meV):

$$f(x) = \frac{ax + b}{2} \times [1 - \mathrm{erf}\left(\frac{E_f - x}{\sigma_{total}\sqrt{2}}\right)] + cx + d$$

$$\sigma_{total} = \sqrt{(1.7 \times K_b \cdot T)^2 + \sigma^2}$$

$$exp. uncertainty = 2 \times \sqrt{2 \cdot ln2} \times \sigma$$

σ_{total} is the total Gaussian broadening including instrumental and thermal ($K_b \cdot T$) broadening, E_f denotes the energy of Fermi-edge. a, b, c, d are dependent variables in the fit function.

Quantification

All XPS data were fitted and quantified by Winspec (LISE, Université de Paix, Namur), The metallic Ga peak is fitted by an asymmetric (Doniach-Sunjic) profile and the $Ga_2O_{3-\delta}$ and SiO_x features are fitted by a Voigt profile. All spectra are fitted subsequently with the same constraints (distance and intensity ratio of doublet peaks, FWHM, ratio of Lorentzian and Gaussian contribution, etc.). The peak area of all core level peaks are corrected by the transmission function of the electron analyzer:

$$I = I_0 * (0.61 + 0.00021 * (E_x - E_b)$$

I and I_0 denote to the corrected peak area and original peak area, respectively. E_x denotes to the excitation energy (Mg K_α = 1253.56 eV), E_b refers to the binding energy of the core level peak. The quantitative analyses of certain core level peaks or elements in this study are then processed by following equation:

$$\frac{A}{B} = \left(\frac{I_A}{\omega_A} \div \frac{I_B}{\omega_B} \div \frac{\mathrm{IMFP}_A}{\mathrm{IMFP}_B}\right)$$

I_A and I_B denote to transmission function corrected peak area of core level A and B. ω_A and ω_B denote to respective photoionization cross section of core level A and B. IMFP_A and IMFP_B refer to the inelastic mean free path of photoelectrons of core level A and B.

Surface coverage of Ga NPs on SiO_x/Si support

The fitting of Ga 3p and Si 2p core level peaks give some insights on the surface coverage of the SiO_x/Si support by the Ga NPs. The applied σ of Ga $3p_{3/2}$ and Si $2p_{3/2}$ core level are 4.27 and 1.28 ($Å^2$) acquired from NIST database, respectively.[1-2] The IMFP for both, the Ga $3p_{3/2}$ and Si $2p_{3/2}$ photoelectrons from Ga and SiO_x is 19.85 Å.[3-5] The intensity ratio of SiO_x/Si is determined as 0.09 by Si 2p core level peaks of the bare SiO_x/Si support (Fig. S1a), and we assume this ratio is the same in the subsequent fitting of the overlapping Ga 3p/Si 2p core level region (Fig. S1b) – it can be expected to have only (if at all) a minor impact on the results of the calculation even though this assumption is not applicable to other samples. The surface coverage of Ga is calculated by the fitted peak area of Ga 3p peak from metallic Ga and Si 2p peak from non-oxidized Si. In the calculation, we assume the substrate is only observable

in the region without Ga coverage. The dewetting behavior of Ga NPs is expected to diminish the Ga thin layer (≦20 Å) on substrate. For surface oxidized sample, due to the IMFP of Ga 3p and Si 2p core level electrons are at least 2 times larger than the film thickness of $Ga_2O_{3-\delta}$ and SiO_x (≦9 Å), only the peak of metallic Ga peak and non-oxidized Si is taken into account to avoid overestimating the Ga coverage.

Ga-O stoichiometry

The fitting of Ga 3d and O 1s core level peaks was used to derive the stoichiometry of the formed gallium oxide. The applied photoionization cross section (σ) of Ga $3d_{5/2}$ and O 1s core level are 1.61 and 6.36 ($Å^2$) acquired from NIST database, respectively.[1-2] The inelastic mean free path (IMFP) for both, the Ga $3d_{5/2}$ and O 1s photoelectrons are 20.9 and 13.9 Å from Ga and SiO_x, respectively.[3-5]

Determination of $Ga_2O_{3-\delta}$ film thickness

In this study, a simple overlayer model is utilized to discuss the $Ga_2O_{3-\delta}$ layer formation on top of metallic Ga, assuming a mechanism of homogeneous, closed packed oxide film growth.[6] The following equation can be used to calculate the $Ga_2O_{3-\delta}$ film thickness, D:

$$D = \lambda_{i,\mathrm{Ga_2O_{3-\delta}}} \cdot \ln\left[\frac{I_{i,\mathrm{Ga_2O_{3-\delta}}} \cdot \lambda_{i,\mathrm{Ga}} \cdot \mathrm{N(Ga)_{Ga}}}{I_{i,Ga} \cdot \lambda_{i,\mathrm{Ga_2O_{3-\delta}}} \cdot \mathrm{N(Ga)}_{\mathrm{Ga_2O_{3-\delta}}}} + 1\right]$$

$\lambda_{i,\, Ga2O3-\delta}$ and $\lambda_{i,Ga}$ are the IMFP values in $Ga_2O_{3-\delta}$ and metallic Ga, respectively for core level *I* (calculated using the TPP2-M equation[7] with the density and electron configuration of stoichiometric Ga_2O_3 as the absorbing layer – we considered this the best approximation available due to the lack of reliable parameters for $Ga_2O_{3-\delta}$). The $\lambda_{i,Ga2O3-\delta}$ and $\lambda_{i,Ga}$ of Ga 3d photoelectrons are 20.9 and 27.2 Å; of Ga 3p photoelectrons are 19.7 and 25.6 Å; of Ga 2p photoelectrons are 5.3 and 6.3 Å, respectively.[1, 4-5, 8-9] $I_{i,\, Ga2O3-\delta}$ and $I_{i,Ga}$ are the intensities (i.e., areas) of the $Ga_2O_{3-\delta}$ and Ga peak contributions, respectively, derived for the core level *i* (obtained by XPS data fitting, see Figs. S4 and S5). $N(Ga)_{Ga2O3-\delta}$ and $N(Ga)_{Ga}$ are the atomic densities of Ga in Ga_2O_3 (0.038 Atoms per cubic Å) and Ga (0.053 Atoms per cubic Å), respectively.[10] It's noted that the formula for D assumes a uniform, closed capping oxide layer, and thus the discrepancies in the oxide thicknesses calculated using Ga 2p (Table S3) and using Ga 3d (Table S4) are related to the observed incomplete coverage of the Ga, which will cause an underestimation of the layer thickness in both cases, with the effect being more pronounced for the more surface sensitive data. As the oxide coverage of Ga increases, the results of the two calculations converge, with the remaining disagreement possibly attributable to the influence of morphology – i.e., the differing relative surface/bulk contributions of the nanoparticles compared to the smooth layer assumed in the calculation.

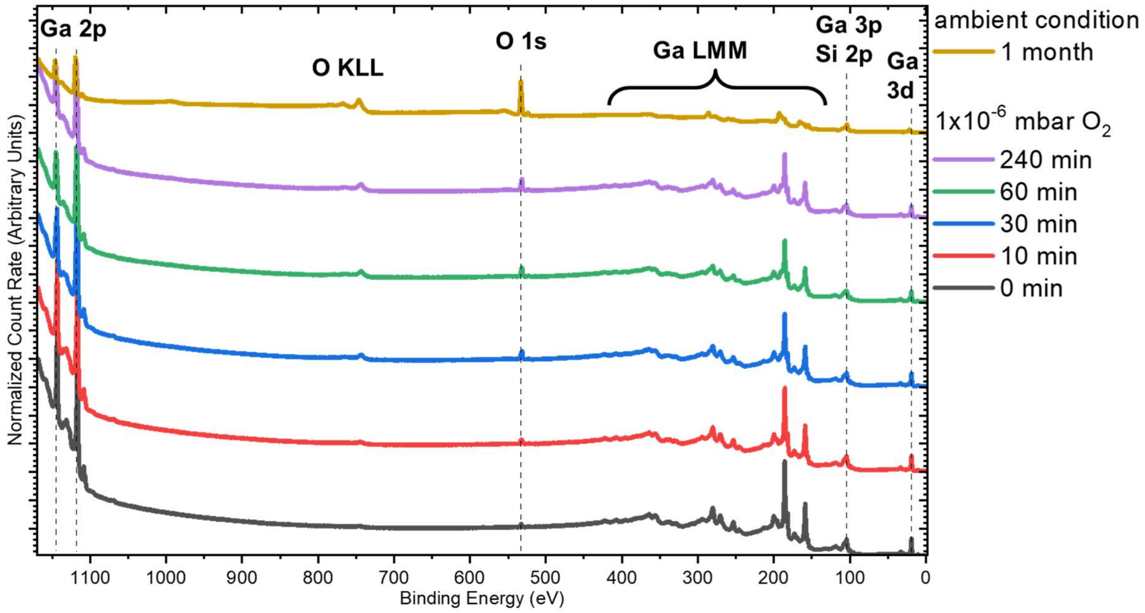

Figure S1. XPS survey spectra recorded with Mg K_a excitation of Ga/SiO_x/Si samples before (black) and after 10 min (red), 30 min (blue), 60 min (green), and 240 min (purple) exposure to 1×10^{-6} mbar O_2. After the 240 min oxidation, the sample was taken out from UHV condition and oxidized at ambient condition for 1 month.

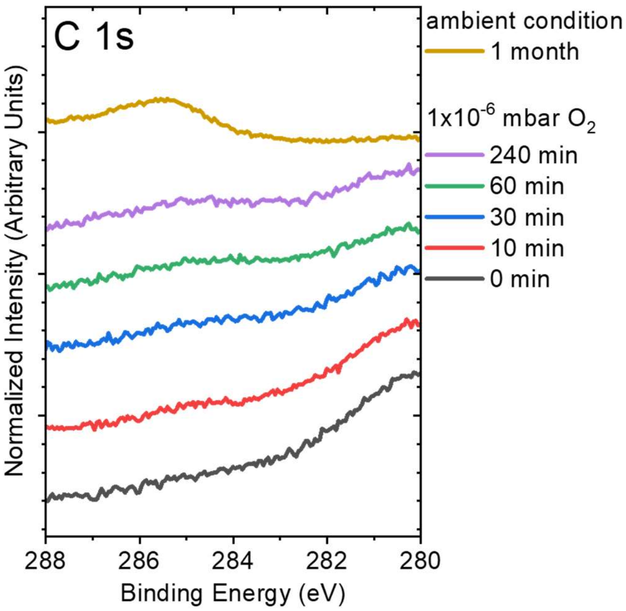

Figure S2. C 1s XPS detail spectra recorded with Mg K_a excitation of Ga/SiO_x/Si samples before and after different oxidation times in 1×10^{-6} mbar O_2. After the 240 min oxidation, the sample was taken out from UHV condition and oxidized at ambient condition for 1 month.

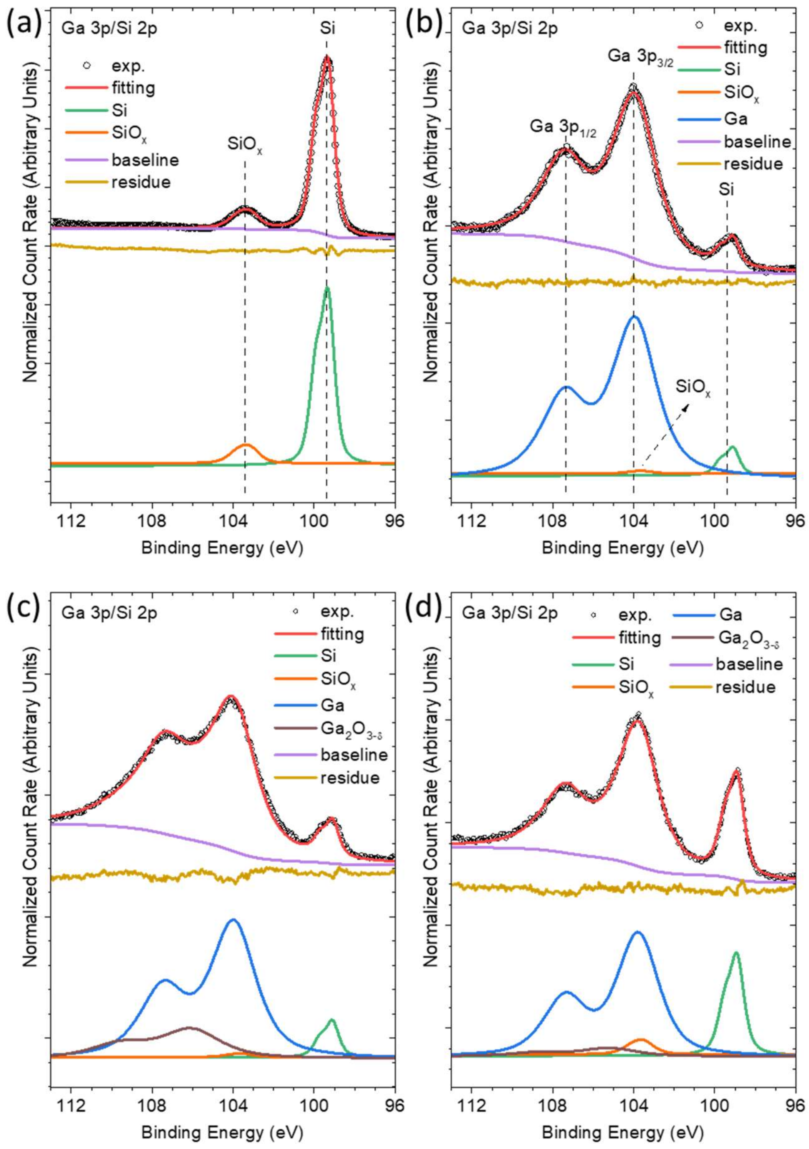

Figure S3. Fit analysis of the Ga 3p and Si 2p peaks of the bare (i.e., not covered) SiO_x/Si support (a) and of a nominal 30 nm thick Ga layer deposited on the same support (b). The evaluation of 30 nm Ga on SiO_x/Si support after 240 mins oxidation in 1×10^{-6} mbar O_2 and after 700 °C annealing in 1×10^{-9} mbar UHV condition is shown in (c) and (d), respectively. The broader peak shape used to fit the $Ga_2O_{3-\delta}$ contribution is tentatively attributed to different oxide environments and/or due to the formed oxide being a less ordered material (compared to the metallic Ga) resulting in varying bond lengths and bond angles – all of which causing BE variations that may increase the FWHM of the Gaussian contribution of the Voigt profile used to fit this spectral component.

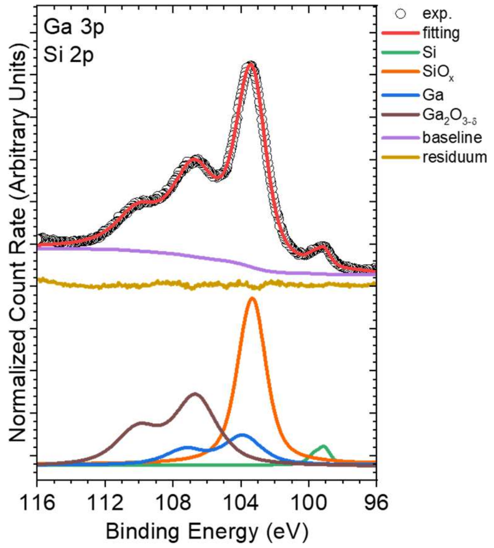

Figure S4. Fit analysis of the Ga 3p and Si 2p peaks of the Ga NPs on SiO_x/Si support after oxidation in ambient condition for 1 month. The SiO_x peak is composed of a spectral contribution of the oxide support and of surface contaminants (presumably a silicate).

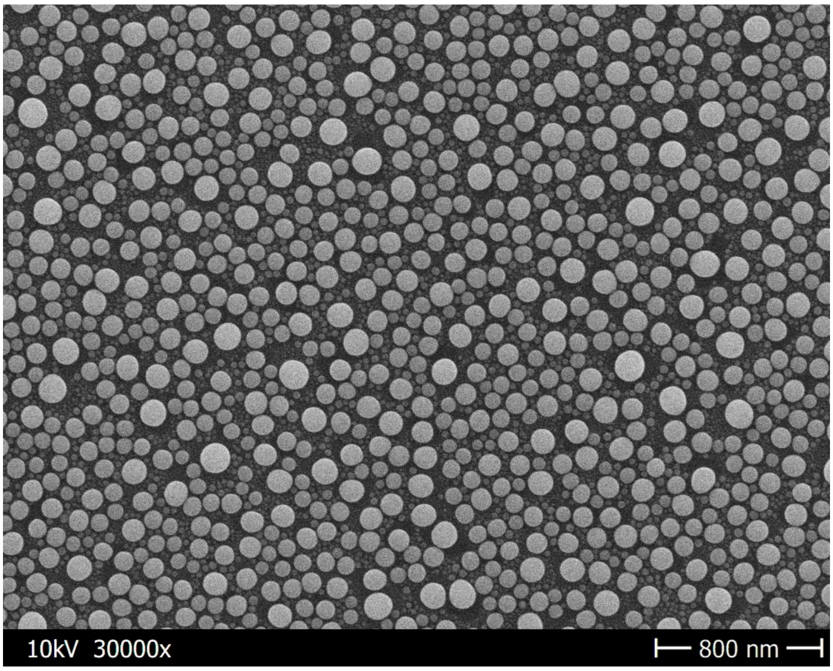

Figure S5. SEM image of Ga nanoparticles on the SiO_x/Si substrate after 240 min oxidation in 1×10^{-6} mbar O_2 and after transfer to the SEM in ambient atmosphere, thus further surface oxidation is expected.

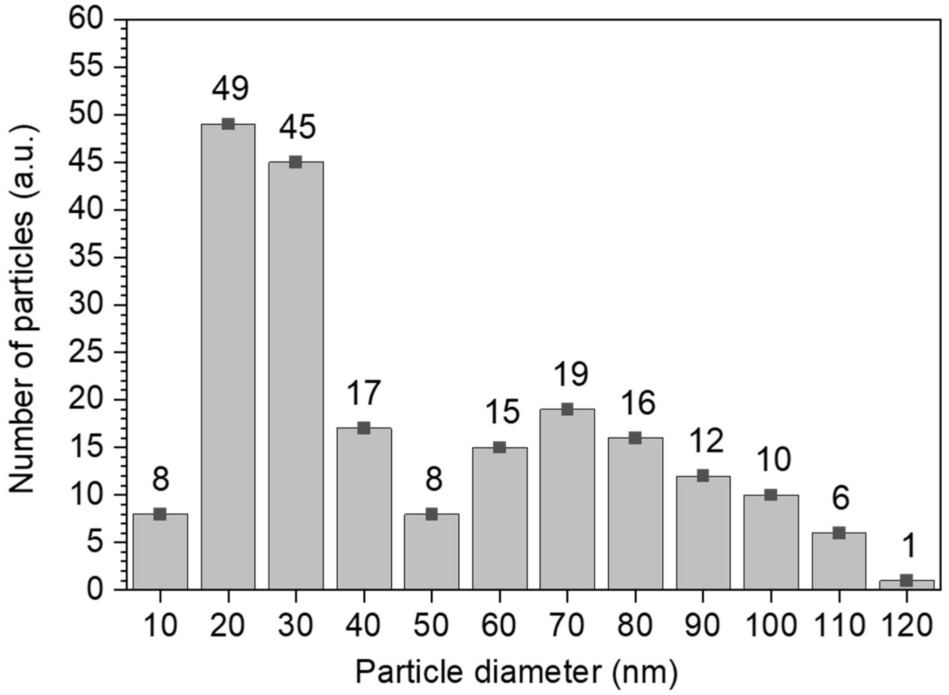

Figure S6. Size distribution of deposited Ga particles based on the statistical evaluation of 206 particles in the SEM image shown in Figure S5.

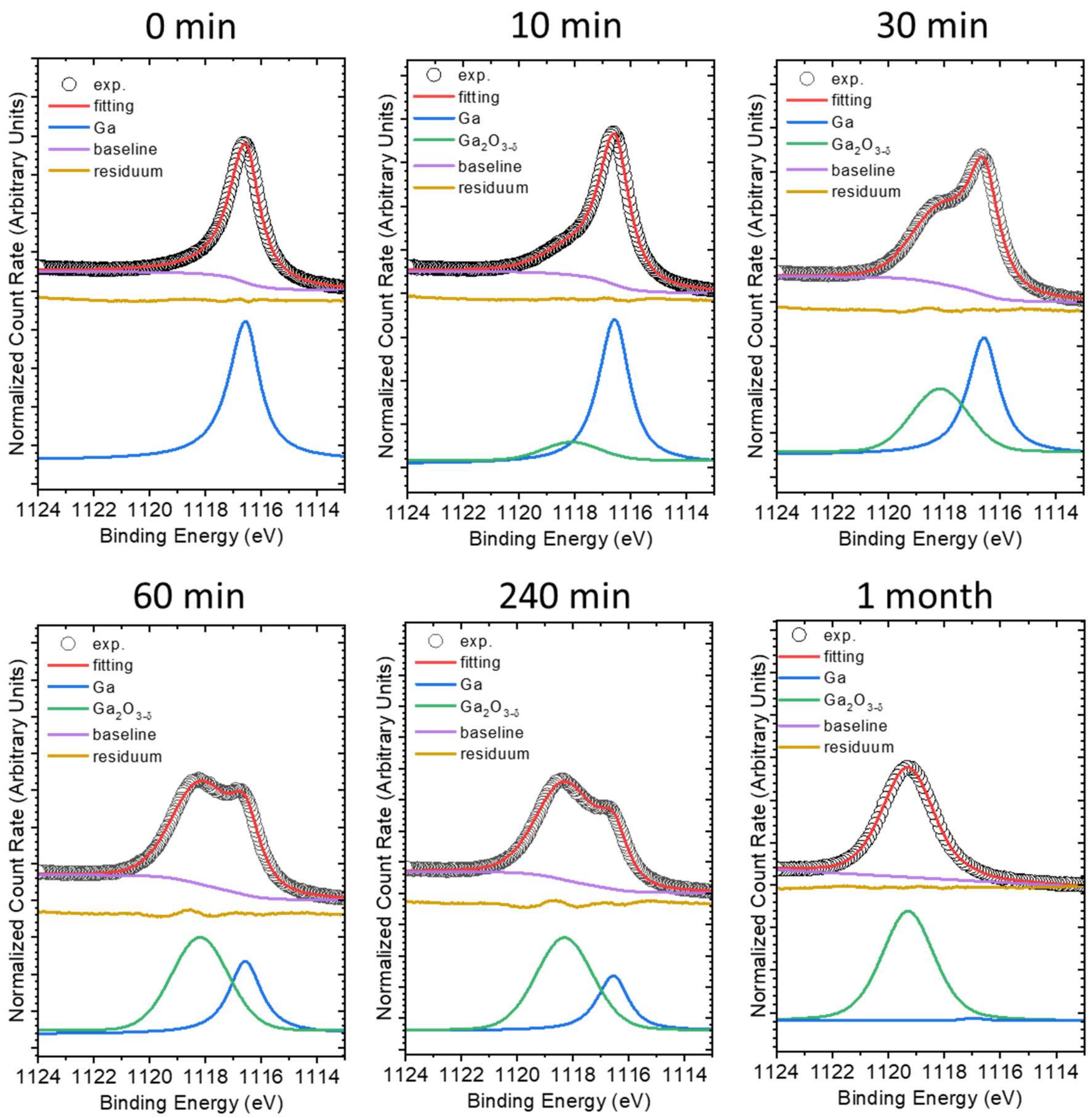

Figure S7. Fitting results of the Ga $2p_{3/2}$ XPS data collected with Mg K_α excitation for $Ga/SiO_x/Si$ samples oxidized in 1×10^{-6} mbar O_2 for different times (0-240 min) and in ambient conditions for 1 month. The metallic Ga peak is fitted by an asymmetric (Doniach-Sunjic) profile, and the $Ga_2O_{3-\delta}$ feature is fitted by a Voigt profile. The broader peak shape used to fit the $Ga_2O_{3-\delta}$ contribution is tentatively attributed to different oxide environments and/or due to the formed oxide being a less ordered material (compared to the metallic Ga) resulting in varying bond lengths and bond angles – all of which causing BE variations that may increase the FWHM of the Gaussian contribution of the Voigt profile used to fit this spectral component.

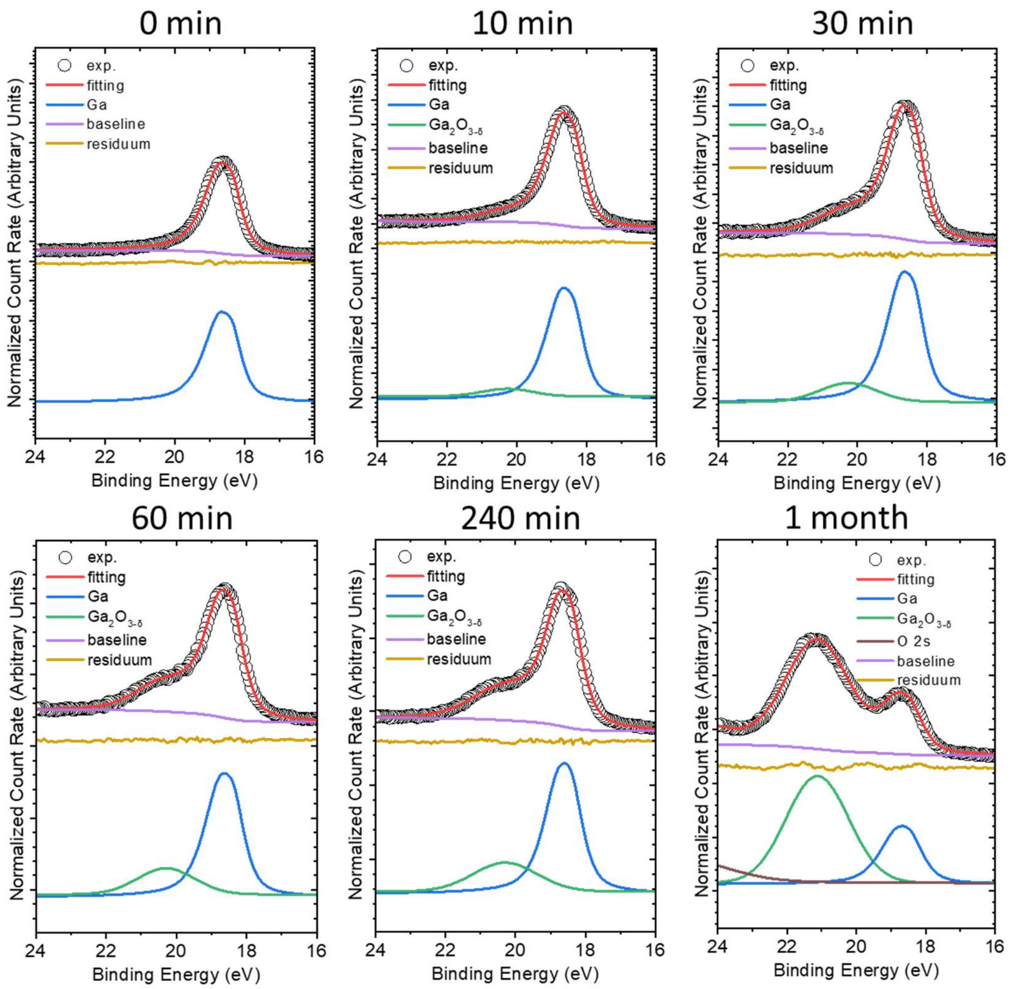

Figure S8. Fitting results of the Ga 3d XPS data collected by Mg K_α excitation for $Ga/SiO_x/Si$ samples oxidized in 1×10^{-6} mbar O_2 for different times (0-240 min) and in ambient conditions for 1 month. Note that the Ga 3d doublet cannot be resolved by the used experimental setup and thus one peak (representing the sum of the Ga $3d_{3/2}$ and Ga $3d_{5/2}$ line) is used to fit each peak contribution. The metallic Ga peak is fitted by an asymmetric (Doniach-Sunjic) profile and the $Ga_2O_{3-\delta}$ feature is fitted by a Voigt profile. The broader peak shape used to fit the $Ga_2O_{3-\delta}$ contribution is tentatively attributed to different oxide environments and/or due to the formed oxide being a less ordered material (compared to the metallic Ga) resulting in varying bond lengths and bond angles – all of which causing BE variations that may increase the FWHM of the Gaussian contribution of the Voigt profile used to fit this spectral component. The high BE background of the spectrum collected for the sample exposed to ambient conditions for one months is dominated by the increased contribution of the O 2s line.

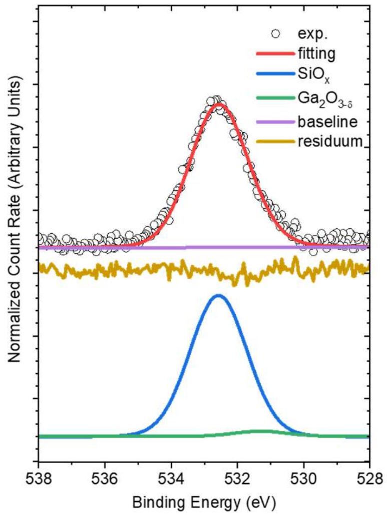

Figure S9. Fitting results of the O 1s detail spectrum recorded with Mg K_α excitation of the as prepared Ga NPs on SiO_x/Si support.

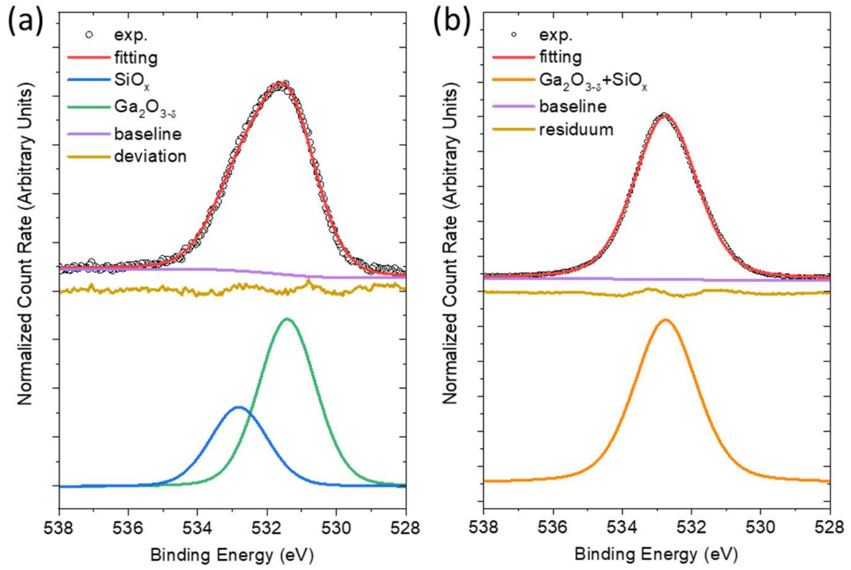

Figure S10. Fitting results of the O 1s detail spectra recorded with Mg K_α excitation of Ga NPs on SiO_x/Si support after oxidation at (a) 1×10^{-6} mbar O_2 for 240 min and (b) in ambient condition for 1 month.

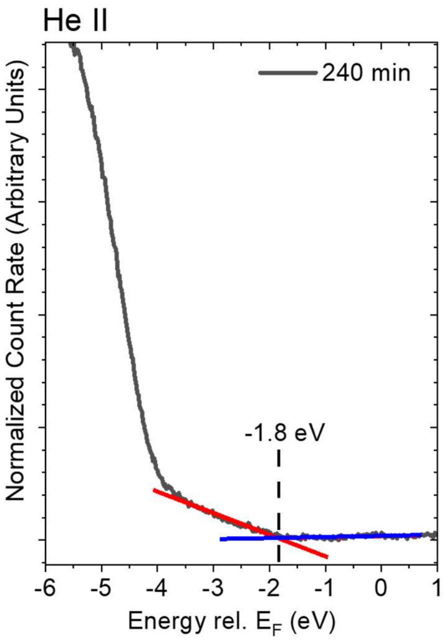

Figure S11. Examination of the above-VBM spectral feature related to oxygen vacancy derived surface defect states in the valence band region. The linear extrapolation is utilized to derive their approximate position with respect to the Fermi level (E_F).

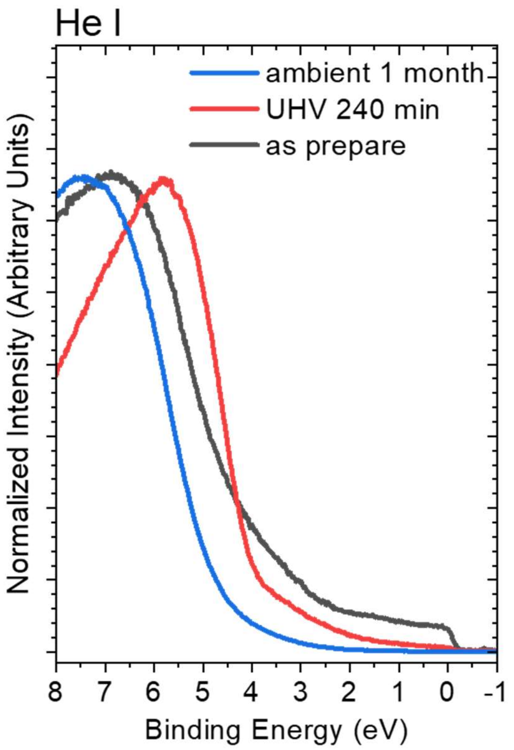

Figure S12. UPS (He I) spectra of Ga NPs on SiO_x/Si support before (black spectrum) and after surface oxidation at 1×10^{-6} mbar O_2 for 240 min (red spectrum) and oxidation in ambient condition for 1 month.

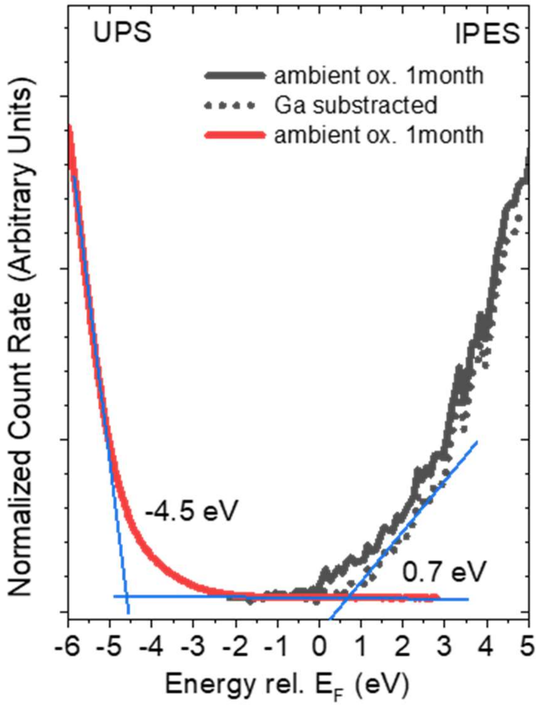

Figure S13. UPS (He II, red) and IPES (black) data (on a common energy scale with "0" indicating the position of the Fermi level) of a Ga/SiO_x/Si sample after 1 month oxidation in ambient conditions. The linear extrapolation to derive valence band maximum (VBM) and conduction band minimum (CBM) positions, respectively, together with the derived values are also indicated. The VBM and CBM values were derived using the UPS and IPES spectra from which the metallic Ga contribution had been subtracted (dashed lines) and have an experimental uncertainty of ±0.1 and ±0.2 eV, respectively.

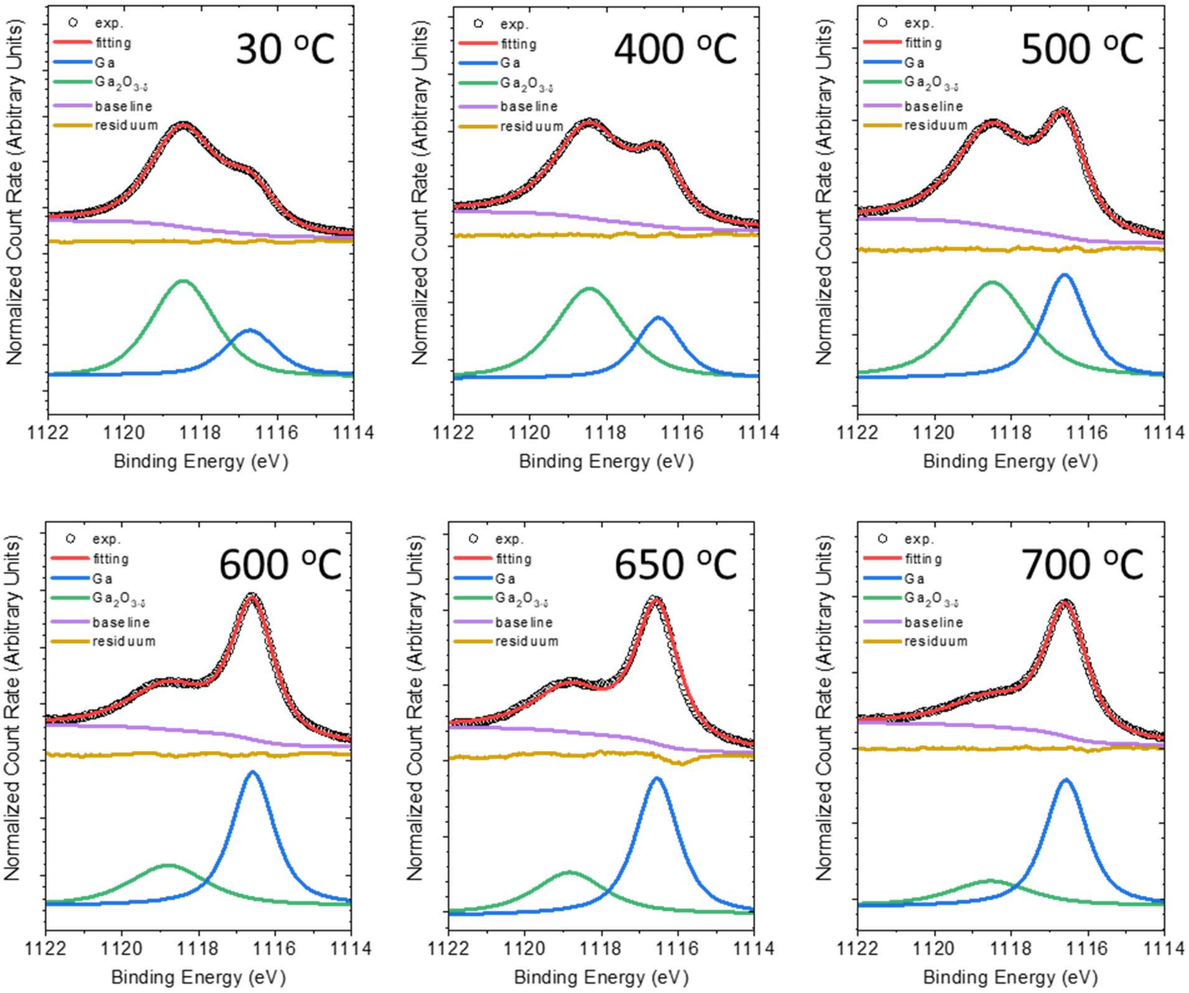

Figure S14. Fitting results of Ga $2p_{3/2}$ peak of Ga NP on SiO_x/Si substrate that had been oxidized at 1×10^{-6} mbar O_2 for 240 min before and after annealing in UHV (1×10^{-9} for 30 min) at temperatures between 400 and 700°C. The metallic Ga peak is fitted by an asymmetric (Doniach-Sunjic) profile and the Ga_2O_3 . . feature is fitted by a Voigt profile. The broader peak shape used to fit the $Ga_2O_{3-\delta}$ contribution is tentatively attributed to different oxide environments and/or due to the formed oxide being a less ordered material (compared to the metallic Ga) resulting in varying bond lengths and bond angles – all of which causing BE variations that may increase the FWHM of the Gaussian contribution of the Voigt profile used to fit this spectral component.

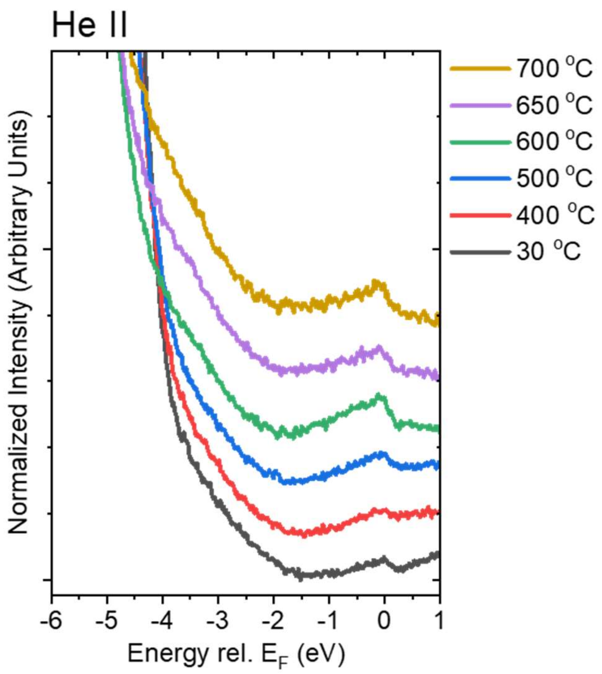

Figure S15. UPS (He II) data of the valence band (VB) of a Ga/SiOx/Si sample oxidized in 1×10^{-6} mbar of O_2 for 240 min before and after annealing in UHV at different temperatures (400-700°C). All measurements were taken at room temperature (i.e., after sample cool down).

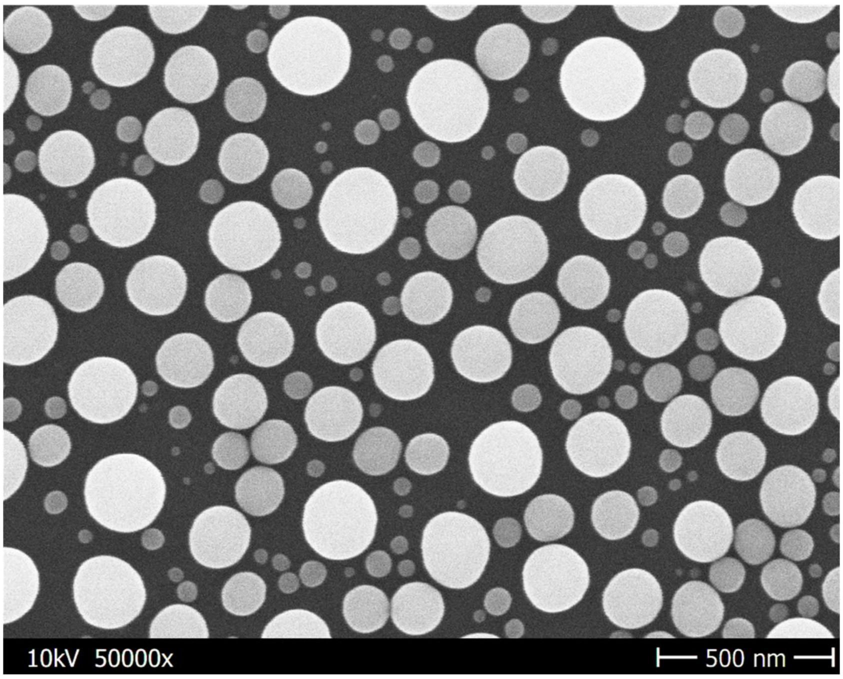

Figure S16. SEM image of Ga NP on SiO_x/Si substrate that had been oxidized in 1×10^{-6} mbar of O_2 for 240 min after annealing at 700 ^oC for 30 min in UHV conditions (1×10^{-9} mbar).

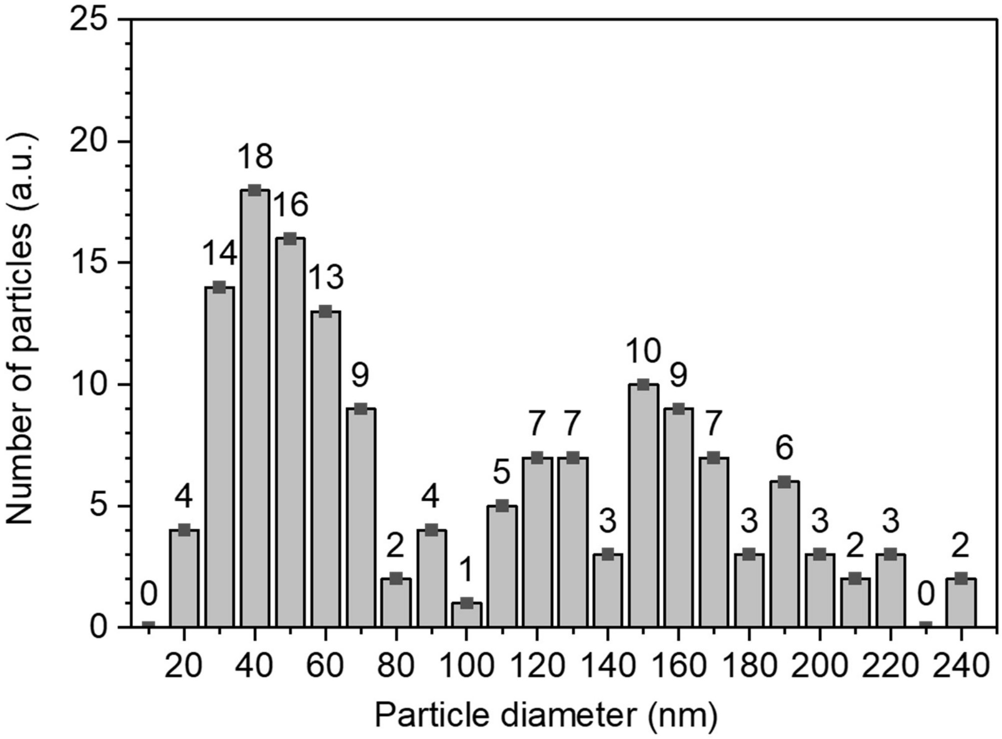

Figure S17. Size distribution of the Ga particles derived by statistical evaluation of 148 particles exhibited in the SEM image depicted in Fig. S16.

Table S1. Analyzer transmission function corrected peak area of the Ga 3p and Si 2p core level peaks of Ga NPs on SiO_x/Si support after different treatments steps. The molar ratio of Ga/Si and surface coverage of Ga NPs on SiO_x/Si support are derived by correcting the peak area by the respective photoionization cross sections (σ)[2] and the inelastic mean free path (IMFP)[4-5, 8]. The fitting results of Ga 3p/Si 2p core level peaks are shown in **Fig. S3-4**.

Status	Core level	Peak area (a.u.)	σ (a.u.)	IMFP (Å)	Ga/Si ratio	Ga coverage (%)
before oxidation (0 min)	Ga $3p_{3/2}$	23146.1	4.27	19.85	**6.3**	**86**
	Si $2p_{3/2}$	1099.3	1.28	19.85		
after oxidation (240 min)	Ga $3p_{3/2}$	17678.1	4.27	19.85	**4.3**	**81**
	Si $2p_{3/2}$	1217.8	1.28	19.85		
after oxidation (1 month ambient)	Ga $3p_{3/2}$	5932.7	4.27	19.85	**4.4**	**81**
	Si $2p_{3/2}$	400.6	1.28	19.85		
after reduction (700 °C)	Ga $3p_{3/2}$	16861.1	4.27	19.85	**1.2**	**55**
	Si $2p_{3/2}$	4173.7	1.28	19.85		

Table S2. Analyzer transmission function corrected peak area of the $Ga_2O_{3-\delta}$ contributions to the Ga 3d and O 1s core level peaks of the sample oxidized in 1×10^{-6} mbar O_2 for 240 min and in ambient conditions for 1 month. Photoionization cross sections $(\sigma)^2$ and inelastic mean free path (IMFP) values applied for intensity correction are listed. The SiO_x and $Ga_2O_{3-\delta}$ peak contributions are obtained from the fitting result depicted in Fig. S10.

Sample	Core level	Peak area (a.u.)	σ (a.u.)	IMFP (Å)	Ga/O ratio
240 min (1×10^{-6} mbar O_2)	Ga $3d_{5/2}$	1926.4	1.61	20.9	**0.86**
	O 1s ($Ga_2O_{3-\delta}$)	13254	6.36	13.9	
	O 1s (SiO_x)	3792.4	6.36	13.9	
1 month (ambient)	Ga $3d_{5/2}$	2420.4	1.61	20.9	**0.15**
	O 1s	43973	6.36	13.9	

Table S3. $Ga_2O_{3-\delta}$/metallic Ga ratio of the respective spectral contributions to the Ga 2p core level and calculated $Ga_2O_{3-\delta}$ film thickness for the Ga nanoparticles oxidized in 1×10^{-6} O_2 for different time.

Oxidation time	$Ga_2O_{3-\delta}$/Ga ratio in Ga 2p	$Ga_2O_{3-\delta}$ thickness (Å)
0 min	0	0
10 min	0.17	1.3±1.1
30 min	0.68	4.1±1.8
60 min	1.60	6.8±1.4
240 min	2.39	8.6±0.4

Table S4. $Ga_2O_{3-\delta}$/metallic Ga ratio of the respective spectral contributions to the Ga 3d core level and calculated $Ga_2O_{3-\delta}$ film thickness for the Ga nanoparticles oxidized in 1×10^{-6} O_2 for different time.

Oxidation time	$Ga_2O_{3-\delta}$/Ga ratio in Ga 3d	$Ga_2O_{3-\delta}$ thickness (Å)
0 min	0	0
10 min	0.08	2.9±1.1
30 min	0.20	6.5±1.8
60 min	0.29	8.8±1.4
240 min	0.3	9.0±0.4

Table S5. $Ga_2O_{3-\delta}$/metallic Ga ratio of the respective spectral contributions to the Ga 2p, Ga 3p, and Ga 3d core levels of the sample oxidized in ambient condition for 1 month together with the corresponding film thickness. The peak area is examined by the fitting results shown in Fig. S4 and Fig. S6-7.

Core level	Species	Peak area (%)	$Ga_2O_{3-\delta}$ thickness (Å)
Ga $2p_{3/2}$	Ga	0.2	36±0.8
	$Ga_2O_{3-\delta}$	99.8	
Ga 3p	Ga	27.4	35±0.8
	$Ga_2O_{3-\delta}$	72.6	
Ga 3d	Ga	28.5	36±0.8
	$Ga_2O_{3-\delta}$	71.5	

References:

1. Wolfgang, W.; Werner, S.; Cedric, P.; Justin, G., Simulation of Electron Spectra for Surface Analysis (SESSA)Version 2.2 User's Guide. Natl Std. Ref. Data Series (NIST NSRDS), National Institute of Standards and Technology, Gaithersburg, MD: 2021.
2. Scofield, J. H., Hartree-Slater Subshell Photoionization Cross-Sections at 1254 and 1487 eV. *J. Electron. Spectrosc. Relat. Phenom.* **1976,** *8*, 129-137.
3. Seah, M. P.; Dench, W. A., Quantitative Electron Spectroscopy of Surfaces: A Standard Data Base for Electron Inelastic Mean Free Paths in Solids. *Surf. Interface Anal.* **1979,** *1*, 2-11.
4. Tanuma, S.; Powell, C. J.; Penn, D. R., Calculations of Electorn Inelastic Mean Free Paths. II. Data for 27 Elements over the 50–2000 eV Range. *Surf. Interface Anal.* **1991,** *17*, 911-926.
5. Tanuma, S.; Powell, C. J.; Penn, D. R., Calculation of Clectron Inelastic Mean Free Paths (IMFPs) VII. Reliability of the TPP-2M IMFP Predictive Equation. *Surf. Interface Anal.* **2003,** *35*, 268-275.
6. Jeurgens, L. P. H.; Sloof, W. G.; Tichelaar, F. D.; Mittemeijer, E. J., Composition and Chemical State of the Ions of Aluminium-Oxide Films Formed by Thermal Oxidation of Aluminium. *Surf. Sci.* **2002,** *506*, 313-332.
7. Tanuma, S.; Powell, C. J.; Penn, D. R., Calculation of Electron Inelastic Mean Free Paths (IMFPs) VII. Reliability of the TPP-2M IMFP Predictive Equation. *Surf. Interface Anal.* **2003,** *35*, 268-275.
8. Tanuma, S.; Powell, C. J.; Penn, D. R., Calculations of Electron Inelastic Mean Free Paths for 31 Materials. *Surf. Interface Anal.* **1988,** *11*, 577-589.
9. Tanuma, S.; Powell, C. J.; Penn, D. R., Calculations of Electron Inelastic Mean Free Paths. V. Data for 14 Organic Compounds over the 50–2000 eV Range. *Surf. Interface Anal.* **1994,** *21*, 165-176.
10. Wittkämper, H.; Maisel, S.; Wu, M.; Frisch, J.; Wilks, R. G.; Grabau, M.; Spiecker, E.; Bär, M.; Görling, A.; Steinrück, H.-P., et al., Oxidation Induced Restructuring of Rh–Ga SCALMS Model Catalyst Systems. *J. Chem. Phys.* **2020,** *153*, 104702.
